# Real time intelligent garbage monitoring and efficient collection using Yolov8 and Yolov5 deep learning models for environmental sustainability

**DOI:** 10.1038/s41598-025-99885-x

**Published:** 2025-05-08

**Authors:** Mohammed M. Abo-Zahhad, Mohammed Abo-Zahhad

**Affiliations:** 1 Department of Electrical Engineering, Faculty of Engineering, Sohag University, Sohag, New Sohag City Egypt; 2 Department of Electronics and Communications Engineering, Egypt-Japan University of Science and Technology (E-JUST), New Borg El-Arab City, Alexandria 21934 Egypt; 3 Department of Electrical and Electronics Engineering, Assiut University, Assiut, 71515 Egypt

**Keywords:** Smart garbage monitoring, Pytorch, Single shot detectors (SSD) model, You-Only- Look-Once (YOLO), Garbage collection, Raspberry Pi, Machine learning, Electrical and electronic engineering, Environmental sciences

## Abstract

Effective waste management is currently one of the most influential factors in enhancing the quality of life. Increased garbage production has been identified as a significant problem for many cities worldwide and a crucial issue for countries experiencing rapid urban population growth. According to the World Bank Organization, global waste production is projected to increase from 2.01 billion tonnes in 2018 to 3.4 billion tonnes by 2050 (Kaza et al. in What a Waste 2.0: A Global Snapshot of Solid Waste Management to 2050, The World Bank Group, Washington, DC, USA, 2018). In many cities, growing waste is the primary driver of environmental pollution. Nationally, governments have initiated several programs to improve cleanliness by developing systems that alert businesses when it’s time to empty the bins. Current research proposes an enhanced, accurate, real-time object detection system to address the problem of trash accumulating around containers. This system involves numerous trash cans scattered across the city, each equipped with a low-cost device that measures the amount of trash inside. When a certain threshold is reached, the device sends a message with a unique identifier, prompting the appropriate authorities to take action. The system also triggers alerts if individuals throw trash bags outside the container or if the bin overflows, sending a message with a unique identifier to the authorities. Additionally, this paper addresses the need for efficient garbage classification while reducing computing costs to improve resource utilization. Two-stage lightweight deep learning models based on YOLOv5 and YOLOv8 are adopted to significantly decrease the number of parameters and processes, thereby reducing hardware requirements. In this study, trash is first classified into primary categories, which are further subdivided. The primary categories include full trash containers, trash bags, trash outside containers, and wet trash containers. YOLOv5 is particularly effective for classifying small objects, achieving high accuracy in identifying and categorizing different types of waste products on hardware without GPU capabilities. Each main class is further subdivided using YOLOv8 to facilitate recycling. A comparative study of YOLOv8, YOLOv5, and EfficientNet models on public and newly constructed garbage datasets shows that YOLOv8 and YOLOv5 have good accuracy for most classes, with the full-trash bin class achieving the highest accuracy and the wet trash container class the lowest compared to the EfficientNet model. The results demonstrate that the system effectively addresses the reliability issues of previously proposed systems, including detecting whether a trash bin is full, identifying trash outside the bin, and ensuring proper communication with authorities for necessary actions. Further research is recommended to enhance garbage management and collection, considering target occlusion, CPU and GPU hardware optimization, and robotic integration with the proposed system.

## Introduction

Garbage, rubbish, junk, and other waste products that people discard often convey a belief that they are useless. Residents in undeveloped nations have expressed worry over overflowing trash bins. The hygienic situation regarding trash control is drastically deteriorating as the population grows. Daily, we witness images of full trash cans with waste spilling out, which causes pollution, or we discover that junk is thrown out of boxes. Garbage is a common source of pollution, including ordinary items such as dry and wet trash, cigarette butts, plastic bags, bottles, and cans. The impact on ecosystems is growing because of these artifacts, whether they are thrown voluntarily or unintentionally. The accumulated trash is a significant environmental issue contributing to resource waste, poor air quality, and water contamination. Additionally, it produces unpleasant smells that are bad for the well-being of living things. It releases poisonous fluids that attract mice, flies, and cockroaches, leading to disorders of the respiratory and digestive systems. Therefore, the solution to the dilemma is to create a system for collecting trash from specific locations to keep cities clean. This gadget monitors the trash cans and tracks whether garbage is thrown inside or outside the bin. An encouraging sound will play if the garbage is inside the box, but if it is outside, a warning sound will play until the person puts the trash back inside the box, at which point the warning sound will cease.

Waste management is becoming increasingly vital as traditional cities transform into smart cities to achieve sustainability, maximize waste efficiency, improve urban mobility, and preserve natural resources. Smart cities will enhance community life through the widespread use of sensors, data supply, and exchange. Scalable, flexible, and reproducible infrastructures are essential as this vision evolves, shaped by various application scenarios and adoption viewpoints. Managing solid waste in smart cities is a significant challenge. Collecting trash from public areas like street corners, parks, or campuses requires additional resources such as personnel and large vehicles to check if bins are full. Zigbee, a traditional wireless technology, offers better physical capacity and level monitoring capabilities. Innovative solutions are crucial for smarter growth and improved urban environments with IoT-based systems. The authors of^2^ propose potential approaches for creating clean, hygienic, and healthy cities by adopting Zigbee. In^3^, intelligent bins connecting IoT devices with garbage collectors are implemented for significant fuel and labor time savings. IoT sensors inside trash cans in public spaces measure the amount of trash present. When the threshold limit is reached, the bins’ status is updated in the cloud to maintain a safe and secure environment. IoT and machine learning (ML) could revolutionize solid waste management. Cyber-physical technologies like IoT and cloud computing present automation opportunities that will transform waste management. In^4^, waste management models published in the literature are thoroughly reviewed, considering IoT requirements. A comprehensive assessment of relevant IoT-based infrastructure for effective waste treatment in urban contexts is conducted^5^. provides a detailed examination of several frameworks for waste management models already used in existing IoT-based infrastructure, effectively treating rubbish while considering IoT needs. These frameworks yield reduced collection times and expenses, fostering citizenship and enhancing the connection between concessionaires and waste producers (citizens).“The following are some of this paper’s significant contributions.

1. Solve the frequent garbage overflow issues in large areas that impact the neighborhoods’ environment and quality of life.
2. Using a Raspberry Pi and a Wi-Fi module, information about the amount of waste is provided, along with various safety precautions when the trash can is fully loaded. So, the system sends a notification to the relevant authority so that additional actions can be taken to empty it.
3. Sensors placed inside the bins continue monitoring how much trash (level and volume) is stored in each bin at any given time. So, the proposed system reduces the need for human intervention and the amount of fuel the collection uses.
4. A camera is placed above the trash bin and connected to a Raspberry Pi to detect if trash is outside the bin. Also, a sensor above the trash is implemented to recognize the level of trash inside the container.
5. A lightweight, accurate, two-stage classification and detection deep learning network based on Yolov5 and Yolov8 was implemented to achieve quick and precise results. It addresses the demands of actual applications while reducing computing costs by considerably decreasing the number of parameters and processes and reducing hardware requirements so that it can be used on embedded systems.
6. A new garbage dataset is constructed from full trash containers, trash bag, trash outside the bins, and wet trash container classes. Each main class is divided into different subclasses.
7. A comparative study of Yolov8, Yolov5, and EfficientNet models on the new garbage and public datasets shows that the Yolov8 and Yolov5 model has good accuracy for most classes, with the full-trash bin class having the highest accuracy and the wet trash container class having the lowest.

The rest of the paper is structured as follows: The literature review of the paper is described in the following section. Waste products and garbage processing are addressed in the Waste materials and garbage processing section. Garbage datasets, including public and newly developed garbage datasets, object labeling, and the proposed model generation, are introduced in the Garbage datasets section. Yolo-based selection of the classification models is explained in the Models selection section. The methodology of the proposed method is described in the following section. This includes the WhatsApp business API with Twilio, playing a warning message, and a Raspberry Pi and web camera connection. The performance metrics for the object detection models are covered in the Performance metrics section. The Results and discussion section explains the results and provides a discussion of the critical findings. The paper’s main conclusions and key findings are summarized in the Conclusion section.

## Literature review

The main objective of most garbage monitoring and collection systems is to design a cost-effective system using technological techniques to pass information to garbage collectors about report container levels and status. Population expansion is the key factor causing environmental problems. Waste management is becoming more challenging for responsible authorities as the population grows. The quick advancement of science and technology may help society handle environmental concerns successfully. IoT, cloud computing, and image processing are cutting-edge technologies that can help the government manage waste and maintain a clean environment^6^. offers a method for achieving waste management when the trash can is full to aid in keeping it clean. The current garbage collection infrastructure and management system are insufficient for the demands of the time. Consequently, there is an excellent facility for collecting trash, which increases the effectiveness of garbage collection. A record is transmitted to the higher authority if the garbage container is not cleaned within a certain amount of time, and they can then take the necessary action against the responsible contractor.

The authors of^7^ described an alternative IoT-based waste monitoring and clearing alarm system. Users can know how much trash is present within each bin, owing to the RGB LED lights mounted to the bins. After the trash has been thrown away, the sensor inside the bin will continue to monitor the level of trash. The local authority will be informed when the garbage level reaches the maximum. If the level is not reached, but the waste has been removed for over two days, a clearance notice will be issued through Wi-Fi. In^8^, the authors accessed cloud data using a Wi-Fi module and an AVR microcontroller for programming. The method keeps the city clean and raises awareness through garbage conditions, utilizing a microcontroller, a few sensors, and a GSM network to deliver short messages. In^9^, a waste management system was developed to track the amount of trash in cans using IoT. Using IoT technologies, an intelligent waste collection system is designed to gather waste data using sensor information and transmit it via a Wi-Fi module^10^.

The authors of^11^ introduce an open IoT stage, an ultrasonic sensor, an LCD screen, a Wi-Fi module, and an Arduino or Raspberry Pi board-based garbage-checking system. The ultrasonic sensor is used to determine the garbage’s depth and estimate the weight of the waste container coming from the heap cell. The LCD screen shows the information, and the Wi-Fi module transmits the data to the web. A comparable IoT-based waste monitoring and clearance alarm system is presented in^12^. An Arduino microcontroller, an ultrasonic sensor, and a Wi-Fi module are all components of this open IoT platform-based trash-checking gadget. The load cell and ultrasonic sensor provide data to the Arduino microcontroller. An ultrasonic sensor is used to measure the depth of the compartment’s garbage, and an estimate of the container’s weight from the heap cell is determined. The authors of^13^ recently developed a robot that moves in a tunnel and performs some operations remotely. The robot is Wi-Fi-controlled wirelessly and equipped with a Raspberry Pi 3 processor that commands the robot’s movement and receives motion orders. Garbage overflow, the most frequent problem in significant locations, has been introduced^14^. This impacted on the local population’s quality of life and the environment. The authors attached an Arduino UNO and a Wi-Fi module to dustbins to provide information about the level of waste and some safety measures in case of fire or rain.

A convolutional neural network (CNN) is the foundation for many object detection methods. The classification of garbage using CNNs has increased to have a high classification accuracy and are small and light. CompostNet^14^, X-DenseNet^15^, and WasNet^16^ are related models. The classification task, however, is significantly influenced by the environment and can only recognize one object type in an image. On the other hand, the detection task may be acknowledged and find several things. The environments and objects can be made more interesting during training, which reduces the influence of the environment and numerous items on the recognition outcomes using object detection models introduced in 2015 by Redmon et al.^17^. Since then, it has gained popularity due to its speed and accuracy. It can predict bounding boxes and class probabilities from complete images in a single network pass, unlike previous models, such as R-CNN. YOLOv8 is the most recent version of the Yolo series, released in 2023 by Ultralytics as a real-time object detector to offer cutting-edge accuracy and speed^18^. It was built on the advancements of previous versions to have new features and optimizations that make it an ideal choice for various object detection applications^19^. De Carolis et al.^20^ and Mao et al.^21^ used an enhanced Yolov3 model for garbage detection. They did not, however, make the network lighter and continued to use much processing. Qin et al. segmented trash images to improve the accuracy of fused segmented trash images with complex backgrounds from other datasets. Then, they trained Yolov3 and Faster Region-based Convolutional Neural Networks (R-CNN) models using the fused data sets^22^-^23^. These studies were created using GPU devices, and as a result, there are many network parameters and heavy computing demands. So, the authors investigated the quickly executed lightweight models while maintaining edge device accuracy.

Contrary to the two-stage detectors, which consider the detection process to be two phases, by first locating the candidate region and then classifying it, one-stage detectors can achieve the results with a single detection. R-CNN, Fast R-CNN, and Mask R-CNN are two-stage detectors, whereas Yolo, Yolov3, Yolov4, Yolov5, and EfficientNet are examples of one-stage detectors. Modern representative object recognition networks were investigated by Zaidi et al., who demonstrated experimentally that two-stage detectors’ inference speeds are far slower than those of one-stage detectors, making it impossible for them to analyze data in real time^24^. Yolov3 and EfficientDet-D2 are among the one-stage detectors that can guarantee real-time and high-accuracy operations, whereas Yolov4 has shown a higher accuracy. In^25^, a new version of Yolov4 offered many noticeable improvements over Yolov3.

The Yolo algorithm can generally process images at a frame rate of 40 to 90 FPS. As a result, it is much faster than alternative methods. This demonstrates that the Yolo algorithm can process a video in real-time with low latency. It is said to be 100 times quicker than R-CNN and 1000 times faster than R-CNN compared to other object identification techniques. Currently, Yolov5 and Yolov8 are state-of-the-art object algorithms developed throughout the years, along with other models to assist in selecting the most appropriate one for garbage classification. Yolov8 is known for its quick processing time, excellent accuracy, and capacity to recognize many objects in a single image. Additionally, it contains a new loss function, a new anchor-free detection head, a new backbone network, and an architecture that makes it simple to compare model performance with earlier models in the Yolo family.

Recently, improper handling of household waste has contributed to environmental pollution and resource depletion, highlighting the need for effective waste sorting^26^. Recent advancements in deep learning and computer vision, such as the YOLOv8 model, offer promising solutions for improving waste detection and classification. However, challenges remain due to the complex nature of waste. The enhanced YOLOv8-CBAM model achieved a mean average precision of 89.5%, demonstrating significant improvements in waste-sorting efficiency and potential applications in smart bins and robotic waste pickers. Moreover, the authors of^27^ developed an optimized YOLOV8 efficient underwater litter detection using deep learning. The optimized YOLOv8s model, fine-tuned through the one factor at a time (OFAT) technique, outperformed other configurations and pre-trained models, showing superior effectiveness and efficiency in various experimental setups. In^28^-^29^, related publications focused on wastewater pollution, caused by organic matter, pesticides, and other contaminants, pose significant environmental risks. These studies propose a data-driven approach to optimize wastewater treatment systems, ensuring the safety and effectiveness of recycled water, particularly for use in coffee plants. Utilizing connected sensors and fuzzy-based data processing with recurrent neural networks, the optimized system enhances water quality monitoring and promotes sustainable agricultural practices by efficiently treating wastewater and enabling its safe reuse.

## Waste materials and garbage processing

**Fig. 1:**
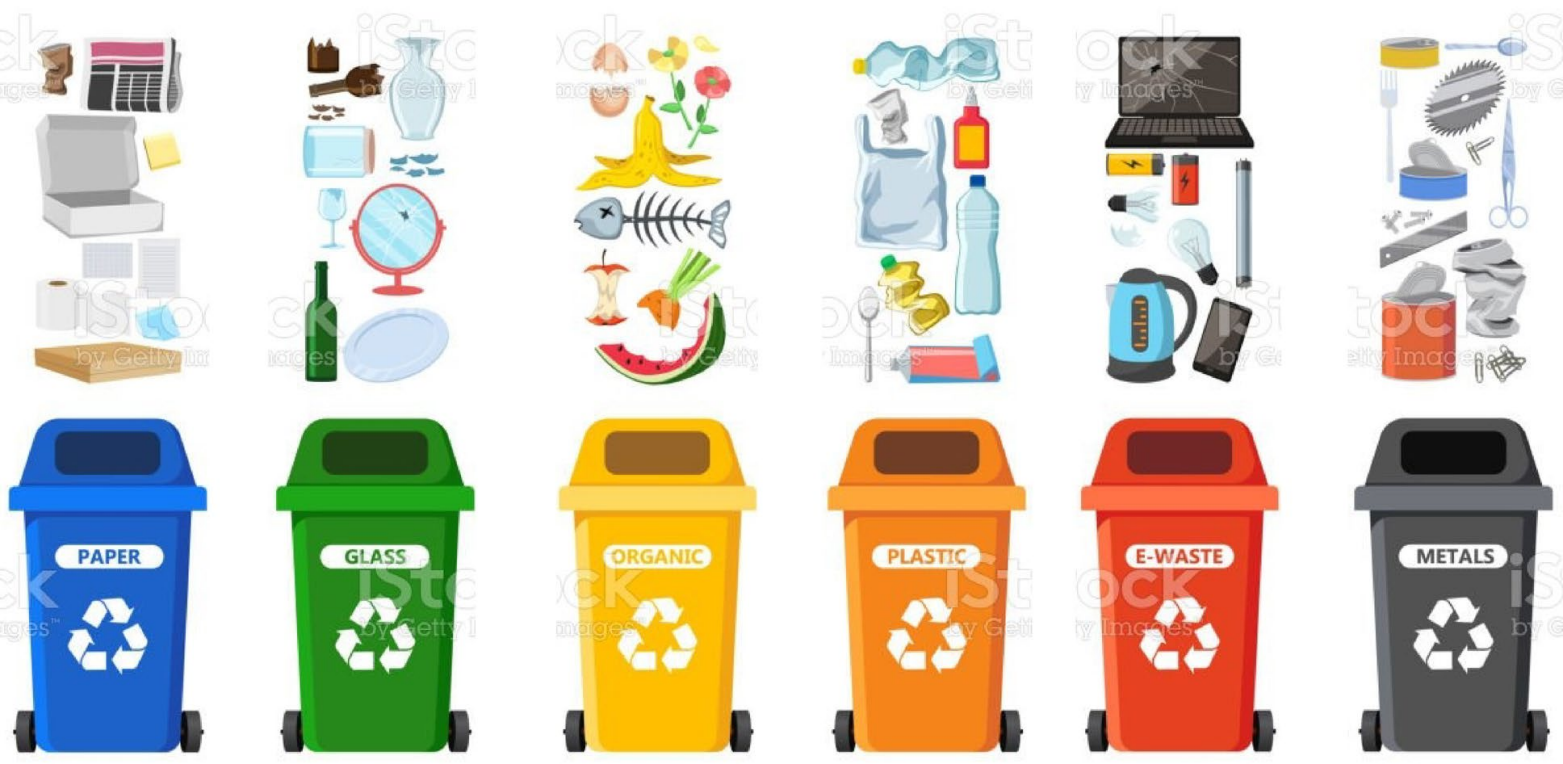
Type of waste materials^5^.

The classification, detection, and recycling of solid waste are the primary approaches to addressing garbage problems. Classification is used to distinguish between different objects, while localization is used to pinpoint the exact location of each object using bounding boxes. Object detection is an effective way to identify and locate specific objects within an image or video. It is essential because it makes selecting objects for collection, recycling, and targeting much more manageable. Selective collection is the cornerstone of efficient waste management and the world’s most widespread recycling technique. Specialized containers for each type of garbage should be taken into consideration since it is essential for an IoT-based waste management system to first classify waste. Industrial, commercial, home, and agricultural garbage can be divided into groups shown in Fig. 1. The several waste types are described as follows:

1. Recyclable waste: This category includes rubbish that may be used to change other parts or create raw materials. It is produced in households, workplaces, and industries, and it needs to be separated so that it may be picked up by designated collection teams and delivered to recycling organizations to complete processing.
2. Organic waste is produced in kitchens, dining establishments, and food-related businesses. It must be differentiated from other waste because it is often intended for city-based trash disposal.
3. Hospital waste: This garbage comes from hospitals and medical facilities. It is handled as carefully as possible and according to accepted best practices.
4. Industrial waste: These are mostly solid residues from the manufacturing process in industries. This substance is typically a residue from raw materials that will be reused in production.
5. Green waste: This describes a particular kind of waste primarily made up of debris that falls to the ground from trees, branches, trunks, and bark trimmings. It is organic and can be decomposed to produce organic fertilizer.
6. Commercial waste is produced commercially by companies selling toys, clothing, and appliances. It is also generated in offices, businesses, colleges, and schools. Nearly all this garbage is recyclable.
7. Commercial garbage is generated in offices, businesses, colleges, and schools.
8. Electronic waste is produced from tossing old consumer electronics that are no longer useful or outdated.

## Garbage datasets

### Public garbage datasets

Waste management strategies include source reduction and reuse, animal feeding, recycling, composting, fermentation, landfills, incineration, and land application. Collection, transportation, treatment, and disposal of trash, as well as monitoring, are just a few of the procedures and operations needed to manage waste from its creation to its final disposal. The five Rs of waste management—reduce, reuse, recycle, and residual management—can enable society to accomplish that. Here, the main objective is to reduce the risks to the environment and human health caused by the improper disposal of waste and resource pollution of the land, sea, and air. Intelligent waste management uses technology and data to make the waste sector more effective. In this case, it is intended to improve resource allocation, lower operating costs, and improve the sustainability of waste services. Worldwide, 78 waste datasets were provided by thousands of users and organizations.

Here, we used the dataset that Thung and Young created for TrashNet^30^. This dataset has 2527 total images distributed over six categories: namely, cardboard, glass, metal, paper, plastic, and trash. The original dataset has been uploaded to Google Drive because it’s about 3.5 GB. Table 1 shows the number of images for each class. About 80% of the data from each category was utilized for training, and 20% was used for testing.

**Table 1:** TrashNet dataset classes and each class’s total number of images.

Class Type	Cardboard	Glass	Metal	Paper	Plastic	Trash
Total Number of Images	403	501	410	594	482	137

### A new developed garbage dataset

**Fig. 2:**
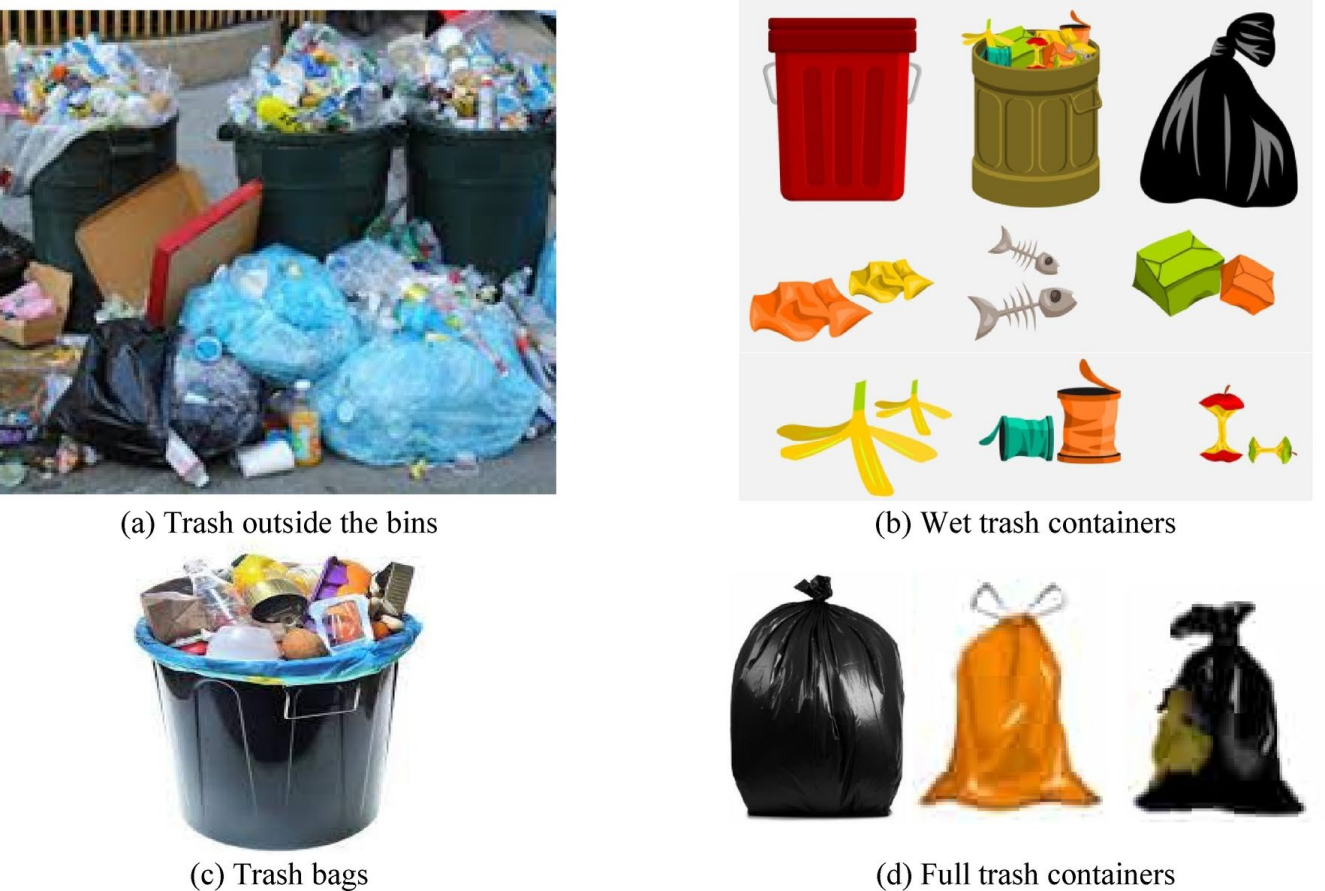
Examples of full trash containers, trash outside the bins, trash bags, and wet trash container classes.

In some countries, waste is broken down into four primary categories: recyclable waste, harmful waste, wet waste, and dry waste, each including various types of garbage. In this case, the trash classification categories do not correspond to the few categories in the Trash Net data set. In this paper, we gathered a collection of garbage images according to the necessary standards to be consistent with the actual situation in Egypt. Consequently, the author constructed a new dataset containing all the data needed for the experiments. A total of 3000 images were used and divided randomly into 80% images for training, and the other 20% were used for testing. Among the images, 1000 were collected from the internet, and 2000 were captured from messy garbage dump areas representing the four classes. This paper considers four classes: full trash containers, trash outside the bins, trash bags, and wet trash containers. Examples of the four classes are shown in Fig. 2. Table 2 includes the number of images for each class in the constructed trash dataset.

Here, we investigated how the training sample size affects the classification model’s accuracy. Deep-learning models can accurately recognize images, which cannot be accomplished without a massive amount of data. Data augmentation is the most effective approach to adding additional images to a dataset and making it as diverse as possible^31^. Consequently, data augmentation is an increasingly common approach to expanding the dataset. This process is intended to avoid overfitting and to lessen data scarcity problems. Thus, it strengthens the model’s robustness and improves the training model’s generalizability. The use of data augmentation enhanced the image’s classification model’s generalization. Data augmentation is frequently done horizontally, rotated, or randomly translated using a few pixels. Generally, data augmentation aims to change the images’ morphology by applying operations like flipping, rotation, magnification, cropping, scaling, and deforming. It can also be done by adding noise, blurring, and changing the color techniques. In this paper, equal efforts have been made to implement data augmentation to increase the dataset size by 50% for each class. This includes: (1) randomly rotate the images within a range of -12.5° to 12.5° to account for different orientations, (2) blur effect of up to 2 pixels to simulate slight variations in image quality, (3) horizontal and vertical flipping of images to accommodate potential variations in the appearance, and (4) randomly scaled the images to 416 by 416 pixels to maintain a consistent image size. Collectively, these augmentation methods are intended to enhance the model’s functionality and the diversity of the dataset. In addition, stochastic gradient descent (SGD) was utilized as an optimizer to evaluate the Yolov8, Yolov5, and EfficientNet architectures.

### Object labeling

**Fig. 3:**
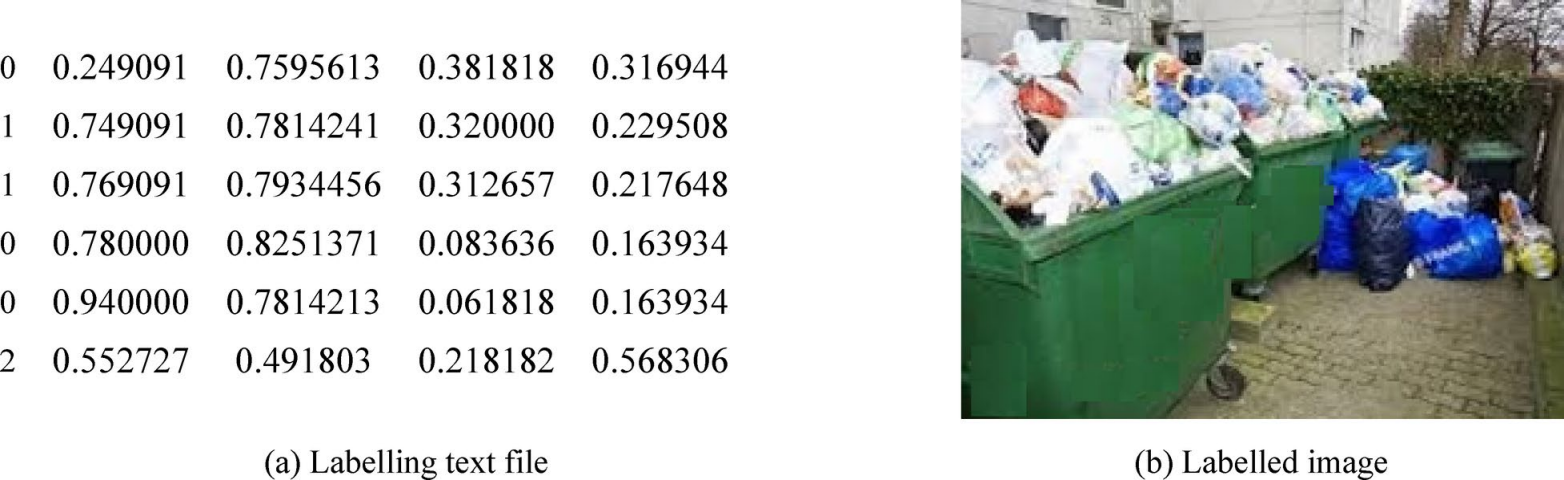
Labeling full garbage containers, wet and trash outside the bins image.

In traditional labeling, images with labels must be made available before the Yolov5 and Yolov8 deep learning models are trained. The model’s performance increases as the labels or annotations get more precise. To construct an annotated dataset for training, we can use the free program Roboflow. The annotation process starts by launching the image and using polygons or bounding boxes to label the data. Then, use Label Assist to create labels automatically and store the data with annotations. This label is recorded as a text file with the labeled object’s class number and the bonding box’s edges, as shown in Fig. 3-a. The model is fed these images and labels to begin training. Using the architecture above, the model starts to forward images while determining the parameters that use stochastic gradient descent to minimize the cost function. An example of the labeling process is illustrated in Fig. 3-b.

Landmarking labeling is also adopted to control the model’s accuracy. The landmarking technique can considerably improve the recognition algorithm, particularly regarding object recognition against varied backgrounds. Since all the background information around the object can be eliminated using this method, the recognition algorithm will only extract information about the object’s features, not its background. The integration of this technique and adopted deep learning methods yields a reasonably accurate detection result. This method allows for the complete removal of the object background, allowing the recognition algorithm to extract only the features of the object itself. Here, a program is written in Python to handle the labeling process of images. This requires changing the traditional labeling method to Yolo Land marking labeling and beginning to surround the objects with an irregular polygon, image by image^32^.

**Table 2:** Developed dataset main classes and the total number of images for each class.

Main Class Type	Full trash container	Trash bag	Trash outside the bins	Wet trash container
Main Class ID	0	1	2	3
Number of Images	912	710	664	714

### Proposed model generation

A Pytorch model is generated, and the Python training code of Yolo is run with selected parameters. The main libraries used to run the code are:

- Open-Source Computer Vision (OpenCV): A free and open-source library of deep learning and computer vision techniques containing over 2,500 optimized image and video analysis algorithms. This library has been used here to take images and capture real-time video from the webcam attached to the proposed system.

- NumPy is a Python library that contains functions for conducting mathematical operations and statistical analysis and Fourier transforms on massive, multi-dimensional arrays and matrices of numerical data.
- PyTorch is an open-source machine-learning library based on the Torch framework. It is utilized for natural language processing and computer vision. This library also offers dynamic computational graphs that allow users to change the graph’s structure and find automatic differentiation for gradient-based optimization.

## Models selection

Target detection is widely used in many industries, such as aerospace, intelligent monitoring, and industrial inspection. Target identification methods have rapidly advanced with the growth of deep learning theory. Most methods are categorized into one-stage and two-stage target detection techniques. The region and, subsequently, the class are predicted by a two-stage network. Two-stage target detection algorithms can find targets by picking candidate regions from the input image and then classifying and localizing them. These algorithms perform well and are highly accurate; however, they also have several drawbacks, including a slow processing rate and a lack of sensitivity to small targets. The one-stage target detection approach directly determines the bounding box’s location and the category that it belongs to through regression to detect targets. It has been noticed that single-stage object detectors provide additional benefits and are simple to integrate with OpenCV.

Liu et al. introduced the Single Shot Detectors (SSD) method in 2016^33^. In contrast to Faster R-CNN, which relies on CNN candidate region extraction for direct detection, SSD uses CNNs. The final forecast is then updated to reflect the detection results. Identical to Yolo, SSD predicts the bounding boxes based on the feature maps of each convolutional layer (the result of each filter or layer). The 3 × 3 convolutional kernel is applied to the combined feature maps to forecast bounding boxes and classification probabilities. SSD is less expensive than two-shot detectors in their depiction. They accomplish comparatively better results in a use case with limited resources.

Yolo is a real-time image-processing method for object recognition that has recently grown more prominent than other algorithms. In Yolo algorithms, CNN is applied to the image and divided into grids. For each grid, bounding boxes. Then, the relevant confidence score is calculated, and the bounding boxes are defined along with the estimated confidence score. In addition to identifying targets, they also get the position of the bounding box and its categorization. This maintains excellent accuracy while accelerating target identification time by reducing the detection problem to a complete set regression issue. Although the Yolo method has strong detection performance, it still needs to recognize most object classes in complicated scenarios with high-speed transitions. As a result, the performance of the target can be enhanced by applying the modifications to the Yolo algorithm, especially in certain areas^34^-^35^. Table 3 illustrates the evolution of Yolo object detection models^36^.

**Table 3:** Evolution of Yolo object detection models^36^.

Year of Publication	2015	2016	2018	2020	2021	2022	2023
Yolo Version	Yolov1	Yolov2 (Yolov 9000)	Yolov3	Yolov4, Scaled Yolov4, PP-Yolo, Yolov5	YoloX, YoloR, YoloS, PP- Yolov2	DAMO Yolo, PP-YoloE, Yolov6, Yolov7	Yolov8

**Fig. 4:**
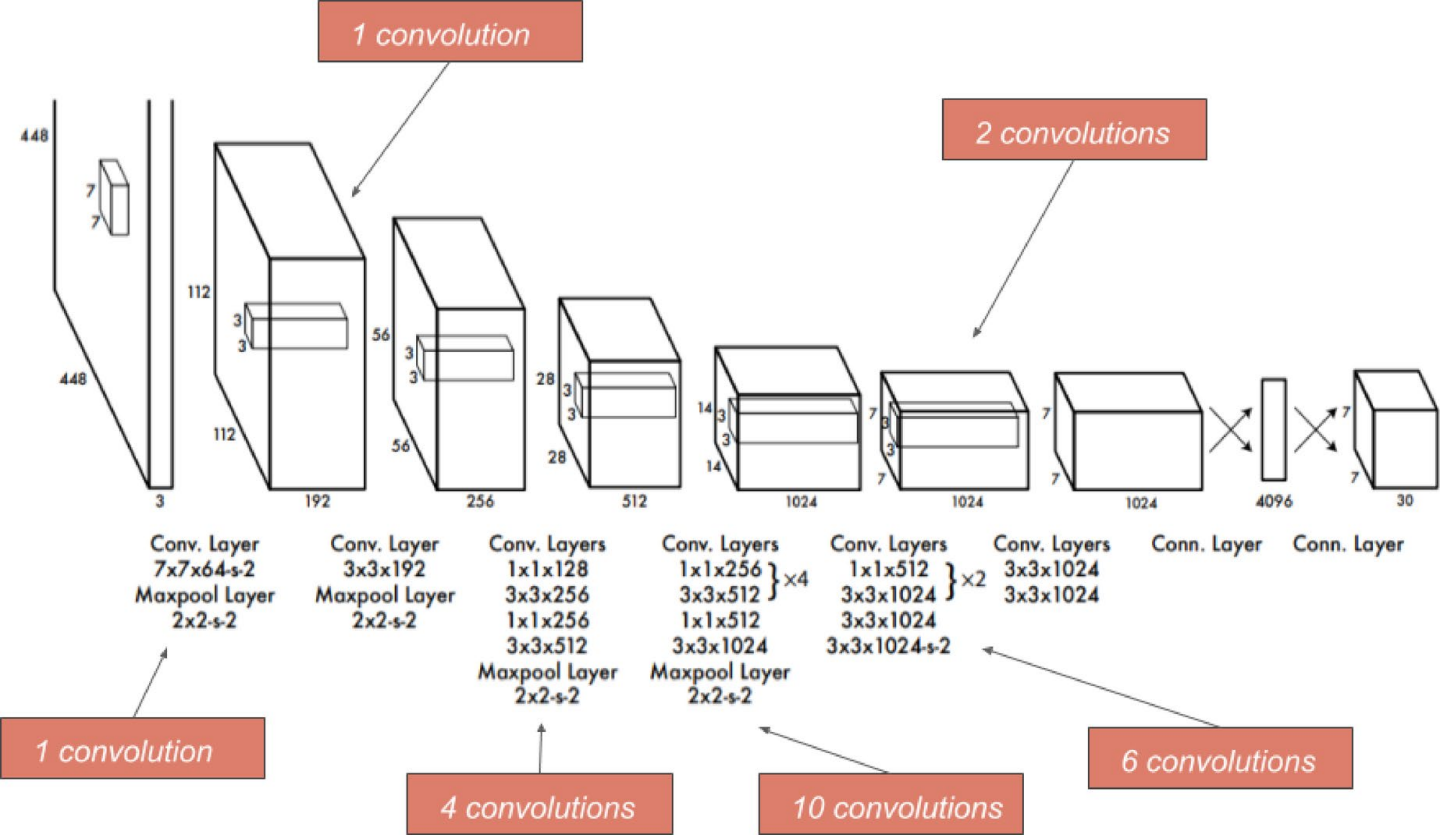
24 Convolution Layers Yolo Architecture^17^.

Figure 4 illustrates the Yolov5 network architecture used as a real-time object detection algorithm^17^. It was developed to run on a single device, such as a laptop or a smartphone, and process frames in real-time, making it suitable for video streams. Thus, Yolov5 is a good choice for real-time object detection tasks where accuracy and speed are essential. However, other machine-learning models may be more suitable for tasks requiring high precision or having more computational resources. In Yolov5, making final predictions requires one forward transit through the network and one glance. The CNN network then transmits this image. This model comprises 24 convolutional layers, four max-pooling layers, and two fully linked layers. To lower the number of layers (channels), we first use 1*1 convolution and then 3*3 convolution. To achieve this, create (1, 1470) from the final ultimately linked layer and resize it (7, 7, 30). The algorithm was trained in two phases: gathering the photos and labeling the items.

Since its creation, the two most often used versions of the Yolo algorithm are YOLOv5 and YOLOv8. These have experienced numerous changes that render them suitable options when accurate small object identification is required. Both variants are excellent in their own right because of their distinct advantages and features. Both algorithms utilize anchor boxes to increase the precision of object detection and suppress repeated detections of the same object, employing non-maximum suppression (NMS). The two approaches employ post-processing to raise the object detection precision and train the model using the Adam optimizer. Yolov5 is known for its object-detecting precision, averaging 50.5% on the COCO dataset. This version of Yolo has excelled in detecting small objects, which have been proven to perform better in practical applications.

**Fig. 5:**
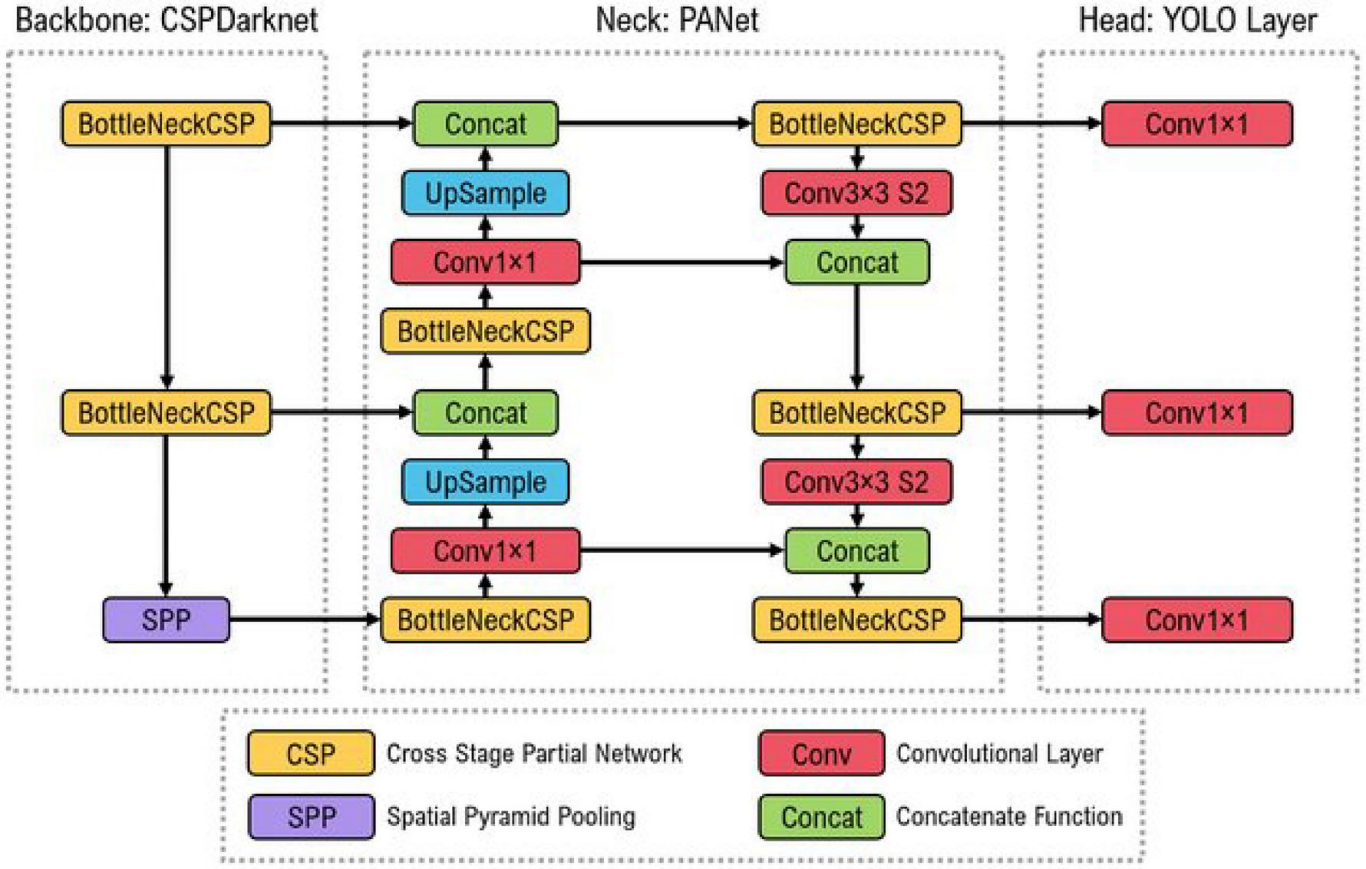
The architecture of the Yolov5 model^39^.

In addition, Yolov5 has an excellent frame rate per second (FPS) and is well optimized for real-time applications. Users of the Yolov8 model should be aware of its limitations. The model may have trouble detecting items in partially obscured or congested scenes. The model might also have difficulty distinguishing small items or objects with low contrast. In contrast, Yolov5 can be a better option if you must deploy a solution on hardware without GPU capability, especially in detecting small objects. It can be considered an improvement over the previous versions in terms of accuracy and speed. Moreover, it has shown excellent results on two official object detection datasets, the Pascal VOC dataset^37^ and the Microsoft Common Objects in Context dataset (COCO)^38^. The COCO dataset, which consists of over 200,000 annotated items grouped into 80 classes, is an excellent choice for training object detection and classification models because it can be employed as a training ground. Figure 5 illustrates the three parts of the Yolov5 model’s construction^39^. The Yolo Layer, PANet, and CSPDarknet represent the system’s head, neck, and backbone. The data is given first to CSPDarknet for feature extraction before being forwarded for feature fusion. Finally, the Yolo layer outputs the detection findings compared to machine learning models.

Table 4 offers an extensive comparison between YOLOv5 and YOLOv8, detailing key differences and justifications presented in the study. This comparison clarifies the strengths, weaknesses, and potential use cases of each model, aiding in determining which model is more suitable for specific applications.

**Table 4:** A comparison between YOLOv5 and YOLOv8.

Feature	YOLOv5	YOLOv8
Detection Precision	High precision for small object detection, averaging 50.5% on COCO.	Improved overall accuracy, outperforms YOLOv5 in specific scenarios. However, it struggles with occlusions.
Speed	Optimized for real-time applications with excellent frame rates (FPS).	Faster in some applications, but less efficient in high-speed transitions.
Architecture	Integrates CSPNet for a reduced model size and lower FLOPS.	Incorporates advanced techniques for feature extraction at the cost of complexity and heavier resources.
Computational Requirements	Lower hardware requirements make it suitable for resource-constrained environments.	Higher computational cost due to added complexity, requiring powerful GPUs.
Training Success	Established performer on the COCO dataset, achieving 50.5% average performance and reaching the desired accuracy quickly.	Newer architecture requires more data and epochs for optimal performance.
Dataset Performance	Perform consistently well across various datasets, including the Trash Net dataset.	Outperforms YOLOv5 on specialized datasets but struggles with diverse conditions.
Model Complexity	Simpler architecture makes it easier to deploy and maintain.	More complex due to additional features that enhance accuracy but require more tuning.
Post-Processing	Employs non-maximum suppression (NMS) for better detection accuracy by filtering out duplicate detections effectively.	It also uses NMS but has been noted to have difficulty detecting in congested, crowded scenes.
Deployment Use Cases	Best for applications needing fast processing on limited hardware.	Suitable for more resource-rich applications suited for cloud-based systems where computational power is not an issue.
Adaptability	It is easier to adapt for various tasks with lower data requirements.	More adaptable to complex tasks but require careful tuning and larger datasets.

EfficientNet, Yolov5 and Yolov8 are adopted in this paper because they perform well for real-time applications and have distinct features regarding hardware requirements. Yolov models offer advantages and disadvantages when selecting the most appropriate object detection model. First, Yolov5 is built by integrating a cross-stage partial network (CSPNet) into CSPDarknet. The basic structure of Darknet (CSPNet) reduces the model’s parameters and FLOPS (floating-point operations per second). This decreases the model’s size while simultaneously speeding up and accurately deducing findings. It is necessary to overcome the issues with repeated gradient information in large-scale backbones and incorporate gradient changes into the feature map. Speed and accuracy are crucial for garbage detection and applications with constrained resources and small model sizes.

Second, to improve information flow, Yolov5 uses the path aggregation network (PANet) as its neck. PANet may enhance the transmission of low-level features by combining an original feature pyramid network (FPN) topology with an improved bottom-up strategy. Additionally, adaptive feature pooling links the feature grid and all feature levels and enables the immediate transmission of each feature level’s essential data to the following subnetwork. The accuracy of the object’s location may be improved in lower layers by PANet’s enhanced usage of precise localization signals. Thirdly, Yolov5’s Yolo layer, which serves as the model’s brain, creates feature maps in three distinct sizes (18 × 18, 36 × 36, and 72 × 72), allowing it to handle tiny, medium, and large objects. Yolov5 is more straightforward to operate; however, Yolov8 is quicker and more precise.

**Fig. 6:**
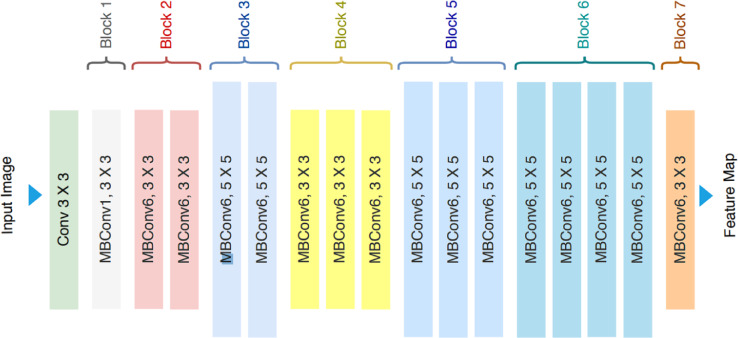
The EfficientNet model architecture (224 × 224 × 3 input image and 7 × 7 × 3 feature map)^40^.

EfficientNet is a series of convolutional neural networks (CNNs) designed for computer vision, introduced by Google AI researchers in 2019. Figure 6 illustrates the model architecture^40^. Its main innovation is compound scaling, which uniformly adjusts the network’s depth, width, and resolution using a single parameter. It is a highly efficient model that combines innovative architectural elements with a compound scaling approach, enabling it to perform exceptionally well in image classification tasks while minimizing the computational overhead. It introduces several innovative approaches to improve efficiency in terms of accuracy per computation.

The compound scaling uniformly scales the network depth, width, and resolution. This strategy contrasts with traditional convolutional neural networks, where typically only one dimension (like depth) is optimized in isolation. Compound scaling allows architecture to expand efficiently by maintaining a balance between the three parameters to maximize accuracy while minimizing resource use. The base architecture of EfficientNet starts from a simple model (EfficientNet-B0), which is constructed using a combination of depth-wise separable convolutions. This type of convolution reduces the computational cost by splitting the convolution operation into two smaller operations, which significantly decreases the number of parameters while retaining important features. Utilizing these two layers of convolutions applies a single filter per input channel, followed by a pointwise convolution that combines the depth-wise outputs. This configuration allows for significant reductions in the number of parameters and computational complexity compared to standard convolutions. EfficientNet incorporates MBConv blocks inspired by MobileNet, where the structure includes a linear bottleneck. In each block, the input is first expanded, processed with depth-wise separable convolutions, and then projected back down to a lower-dimensional space. This design helps capture important features efficiently while keeping computational demands low. The model employs the Swish activation function, defined as f(x) = x⋅sigmoid(x). Research shows that Swish can produce better results than the traditional Rectified Linear Activation (ReLU) function, particularly in deep networks, contributing to enhanced performance in classification tasks. EfficientNet uses a stochastic depth technique, where random layers are skipped during training to improve generalization, and an extensive data augmentation strategy to enhance the model’s robustness against overfitting. This model is often trained on the ImageNet dataset, and the pre-trained weights are adapted for transfer learning on various downstream tasks. This allows for easier adaptation to other image classification tasks with smaller datasets. Due to its architecture and efficient training strategies, EfficientNet achieves state-of-the-art accuracy on image classification benchmarks while using fewer parameters and less computational resources compared to other models like ResNets or DenseNets. It strikes a balance between high performance and efficiency.

The system’s foundation described in the present research is the single-class detector that finds garbage classes in the image. They have demonstrated different accuracies when comparing deep learning algorithms to machine learning and image processing techniques. However, deep learning algorithms, on the other hand, require a considerably larger training dataset to achieve the best accuracy. As a result, we employ pertained networks and fine-tune the final layer considering the input dataset. Transfer learning, which involves freezing the weights of the first layers and only changing the weights of the last layer, is a method for fine-tuning pre-trained networks.

**Fig. 7:**
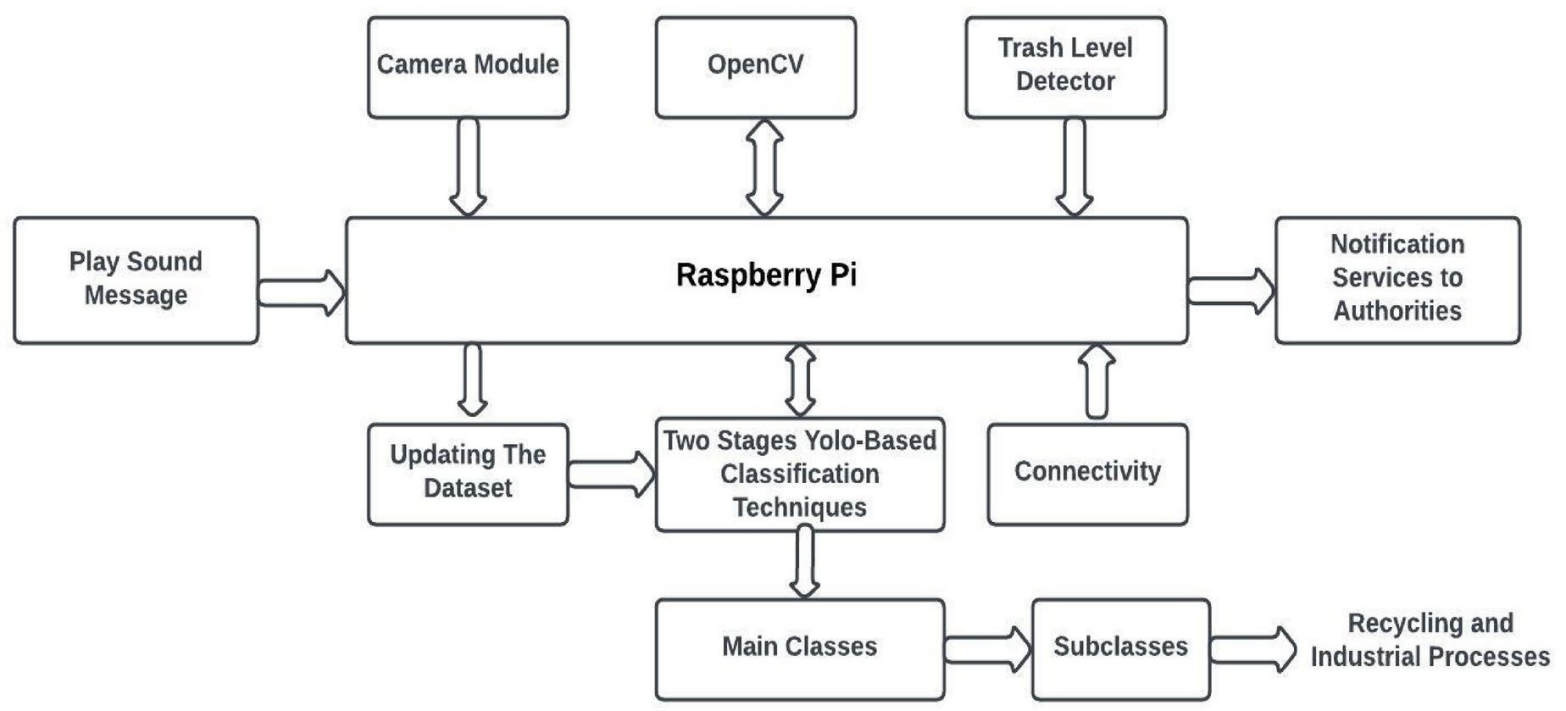
The proposed system architecture.

**Fig. 8:**
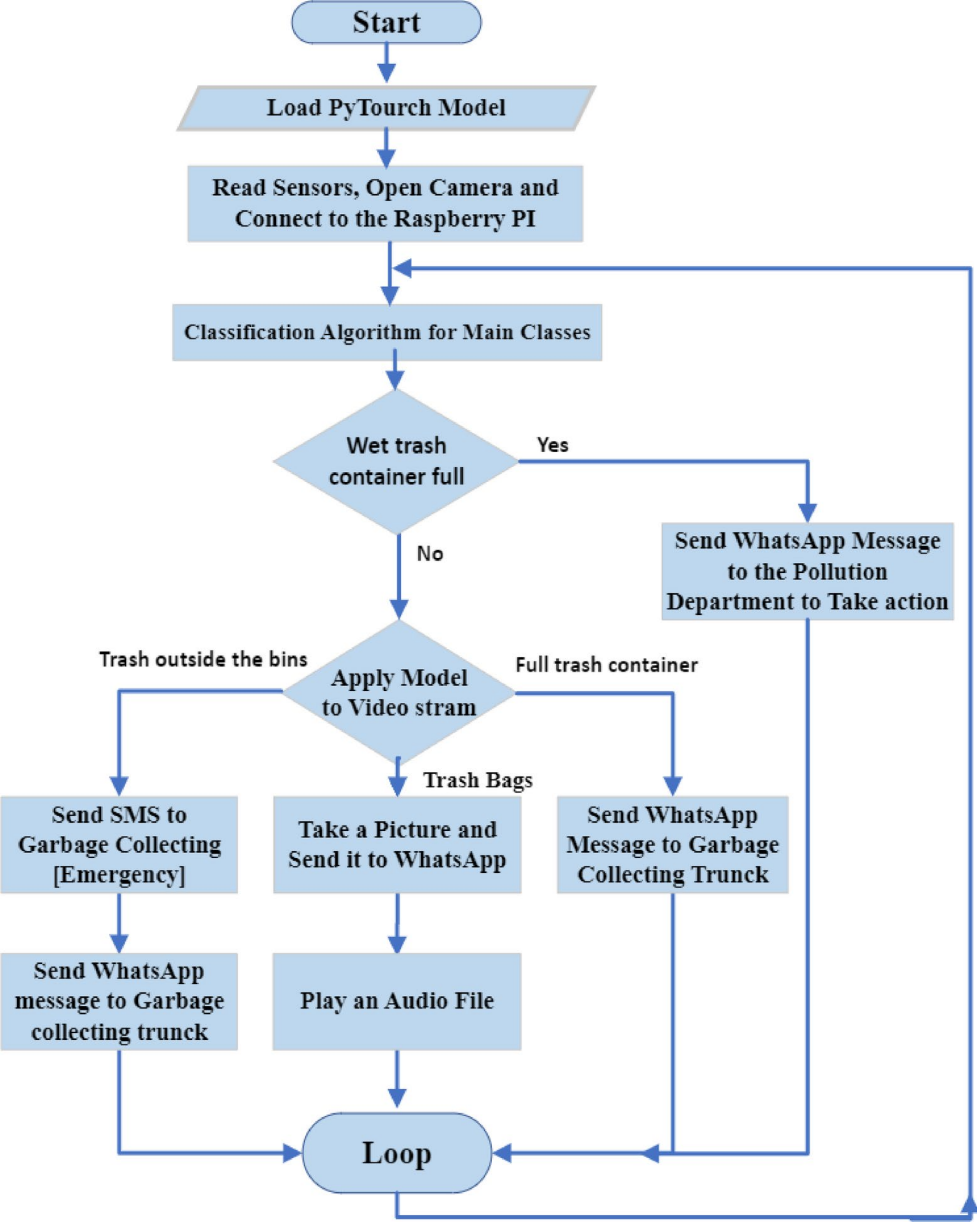
The System Flow Block Diagram.

There are two ways to detect garbage outside the garbage box. The first is when someone throws the trash bags outside the garbage box. In this case, a camera and Raspberry Pi send a photo to the responsible parties. Turn on an audio file that says, “Please put the bag inside the garbage box” only once. The second is when the garbage outside the garbage box is vast because the garbage box is full. In this case, an SMS is sent to the garbage truck to come and empty the garbage box. Moreover, the model can detect a full garbage bin without extra garbage outside the box. In this situation, a text message is sent to the trash truck, requesting that it arrive and complete the task. Figure 7 illustrates the proposed system architecture. The block diagram of the system’s operation is shown in Fig. 8.

## The methodology of the proposed method

### Classification of the main classes and subclasses

Most of the garbage that people of concern produce are organic, frequently seen as wet garbage that causes pollution. Although household and marketplace garbage can be significant in volume and weight and contain non-organic materials like packaging, cans, and plastics, it must be classified and processed. All trash must be disposed of in suiTable 100-litre containers with handles and lids or a regular plastic garbage bag. However, most less-developed countries have no standardization or categorization of garbage. Thus, each main class should be divided into subcategories, as shown in Table 5.

**Table 5:** Examples of subclasses in each main class.

Primary classes and their codes	Examples of Subclasses and their codes
Full trash container (0)	Paper (000), rags (001), glass (002), metal (003), bottles (004), wire residues (005), fishing equipment (006), nets (007), bait boxes (009), wood products (010), packaging material (011), old shoes (012), deck sweepings (013), battery (014), disinfectant (015), plastics (016), ….
Trash bags (1)	Paper (100), books (101), newspaper (102), plastic (103), aluminum cans (104), rubber items (105), packaging (106), fabrics (107), clothes (108), bottles (109), cans (110), plastic (111), wood (112), glass (113), cardboard (114), packaging material (115), ….
Trash outside the bins (2)	Food items (200), used tissues (201), paper towels (202), yard waste (203), soiled food wrappers (204), hygiene products (205), paper towels (206), vegetable peels (207), used tea (208), fruits (209), leftovers (210), coffee grounds (211), ….
Wet trash container (3)	Fruit peels (300), food waste (301), eggshells (302), house sweeping (303), coconut shells (304), hair (305), bone fragments (306), sanitary napkins (307), diapers (308), meat (309), fruit leaf (310), vegetable leaf (311), flowers (312), ….

It can be noticed that one subclass can exist in another main class. However, each subclass should have its unique subclass ID that may be used in classification and recycling. Each subclass ID starts with its primary class ID followed by a two-digit number from 00 to 99, allowing 100 subclasses for each main class. For example, if it is in the full-trash container main class, the subclass code of glass is 002; however, it has a subclass code of 113 if it belongs to the trash bag main class.

**Fig. 9:**
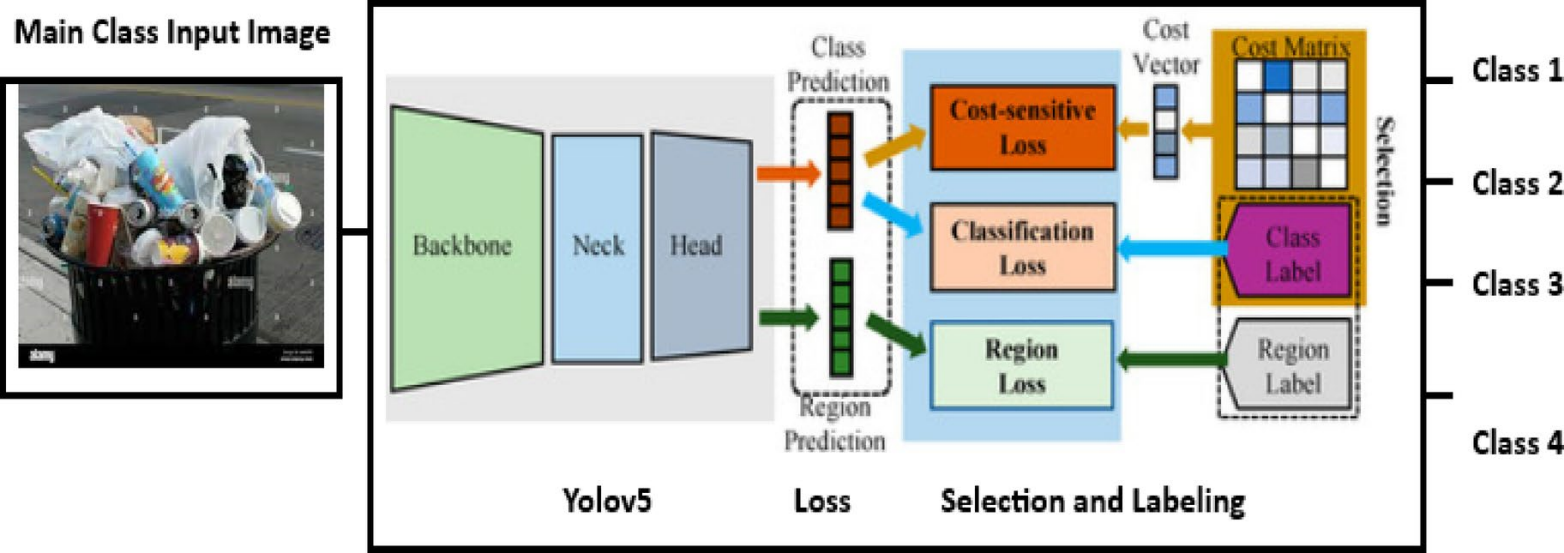
Classification of the main classes using the Yolov5 approach.

**Fig. 10:**
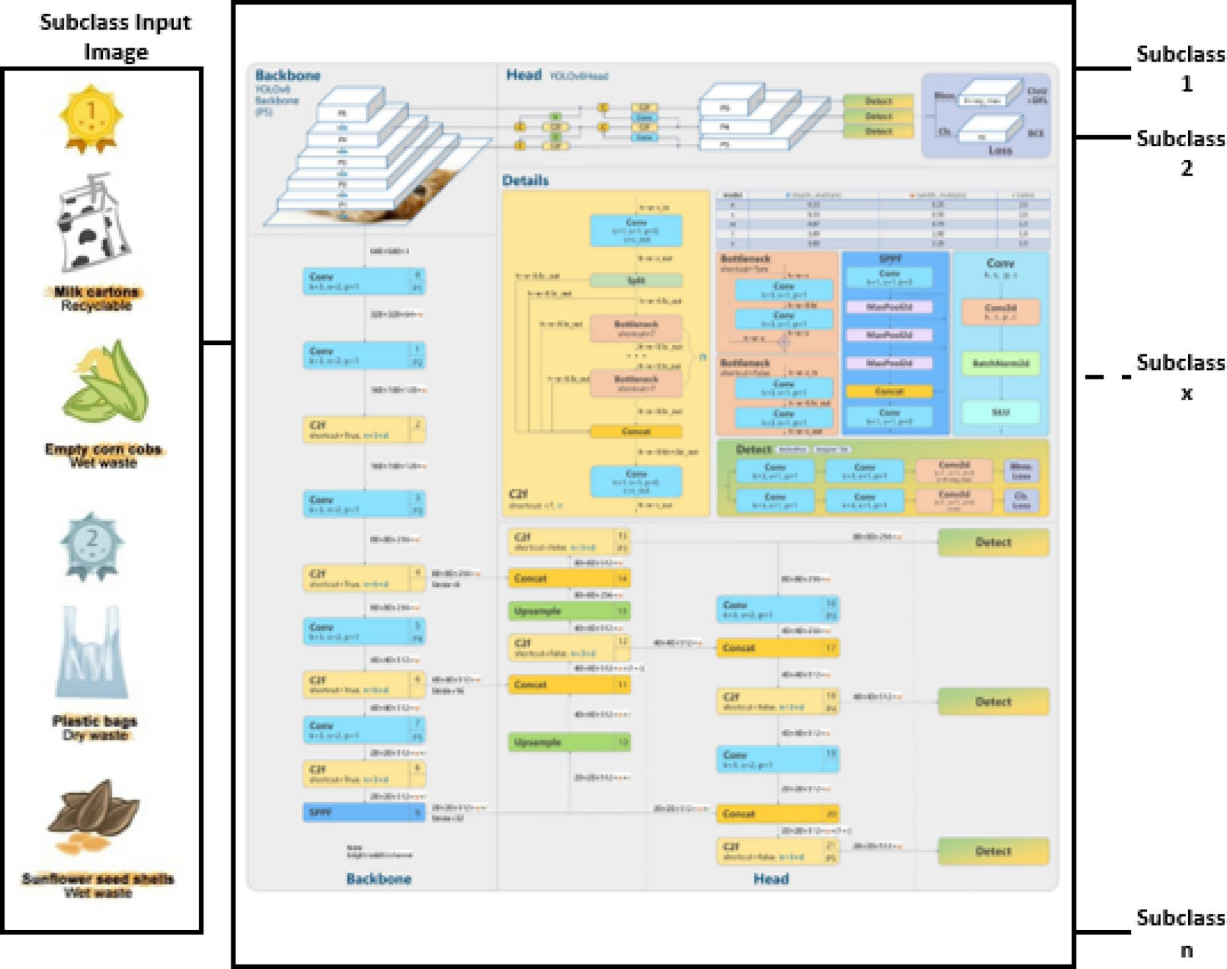
Classification of the sub-classes using the Yolov8 approach.

Here, it is proposed that a two-stage cascaded classification technique, namely Yolov5 and Yolov8, be adopted. The first stage classifies the main classes using Yolov5, followed by Yolov8 as the second stage to classify specific subclasses in their main classes. Figures 9 and 10 illustrate the main classes and subclasses classifications. The classification of the main classes or sub-classes is based on the application. It is enough to categorize the main classes only using Yolov5 if the aim is garbage collection. However, in the case of recycling, the first stage is followed by the second stage based on Yolov8, which must be used.

The reasons for selecting the Yolov5 and Yolov8 models are:

- Both models are preferable for applications that require low latencies. However, Yolov8 is preferable for applications that require real-time object detection.
- The two models are more accurate in object detection tasks than other real-time object detection algorithms.
- Yolov5 is lightweight and designed to run on a single device, such as a laptop or a smartphone, which makes it suitable for applications that require low computational resources.
- Yolov8 is the most recent Yolo version based on the advancements of previous versions for real-time object detection to offer cutting-edge accuracy and speed. It introduces new features and optimizations, making it an ideal choice for various applications.

### The whatsapp business API with Twilio

WhatsApp is the most popular application in many parts of the world. Using Twilio, we can reach more than 1.5 billion users. Building, scaling, and managing real-time communications within applications is made possible by the cloud communications platform Twilio. It provides developers various application programming interfaces (APIs) to integrate voice, video, message, and authentication functions into their programs. To send programmed messages, we need a phone number. Twilio extracts a phone number for the country that the user wants. So, it is necessary to define the numbers to which the programmed messages will be sent to define WhatsApp on Twilio. Then, send this programmed message to the number Twilio extracted through Python code. The programmed messages can be text messages or images.

### Playing a warning message

When someone is being watched, they act correctly and follow the rules. This is the psychological part used by the designed application. If the system detects a trash bag appearing suddenly, someone just threw it outside the trash bin. The system played a prerecorded message via a speaker: “Please put the trash inside the bin.” The results obtained of the system prototype show that, in most cases, this works well, and people will not throw it outside of the bin.

### Raspberry Pi and camera connection

The proposed Pytorch model and Python code are uploaded to a Raspberry Pi attached to a camera. Two widely used approaches function with little setup. Utilizing a camera module compatible with Raspberry Pi is the first choice. The advantages of this approach include excellent support, a wide range of available modules, and the fact that most of the camera-based Raspberry Pi software supports the internal camera interface right out of the box. Such camera modules’ short wire lengths might be a drawback. On the other hand, USB cameras often have a longer cord and decent image quality. However, support cannot always be assured. By utilizing this technique, you can attach numerous USB cameras to a single Raspberry Pi.

## Performance metrics for the object recognition models

The accuracy, precision, recall, precision-recall curve, F1 Score, average precision (AP), mean average precision (mAP), and frames per second (FPS) are used to measure and evaluate the performance of deep learning object recognition models. Moreover, the confusion matrix and the label correlogram are typically used to assess the performance of the classification models. The proposed system’s accuracy is obtained by inserting batches of images into the model and allowing it to detect full trash containers, trash outside the bins, trash bags, and wet trash container classes in the image. A correlogram is a collection of two-dimensional histograms that compare one data axis with another. The labels in the image are in the format xywh, where w and h denote the width and height, respectively. The correlation between labels is displayed on the correlogram according to one of the correlation measures, such as Spearman’s rank correlation coefficient or Pearson’s correlation coefficient.

Pearson’s correlation coefficient (r) determines the linear relationship between the two variables. It is calculated using the following equation:

1 $$r=\frac{{\sum {\left( {x - \overline {x} } \right)\left( {y - \overline {y} } \right)} }}{{\sqrt {\sum {{{\left( {x - \overline {x} } \right)}^2}{{\left( {y - \overline {y} } \right)}^2}} } }}$$

where x and y are the values of the two variables, and x̄, and ȳ are their mean values.

**Fig. 11:**
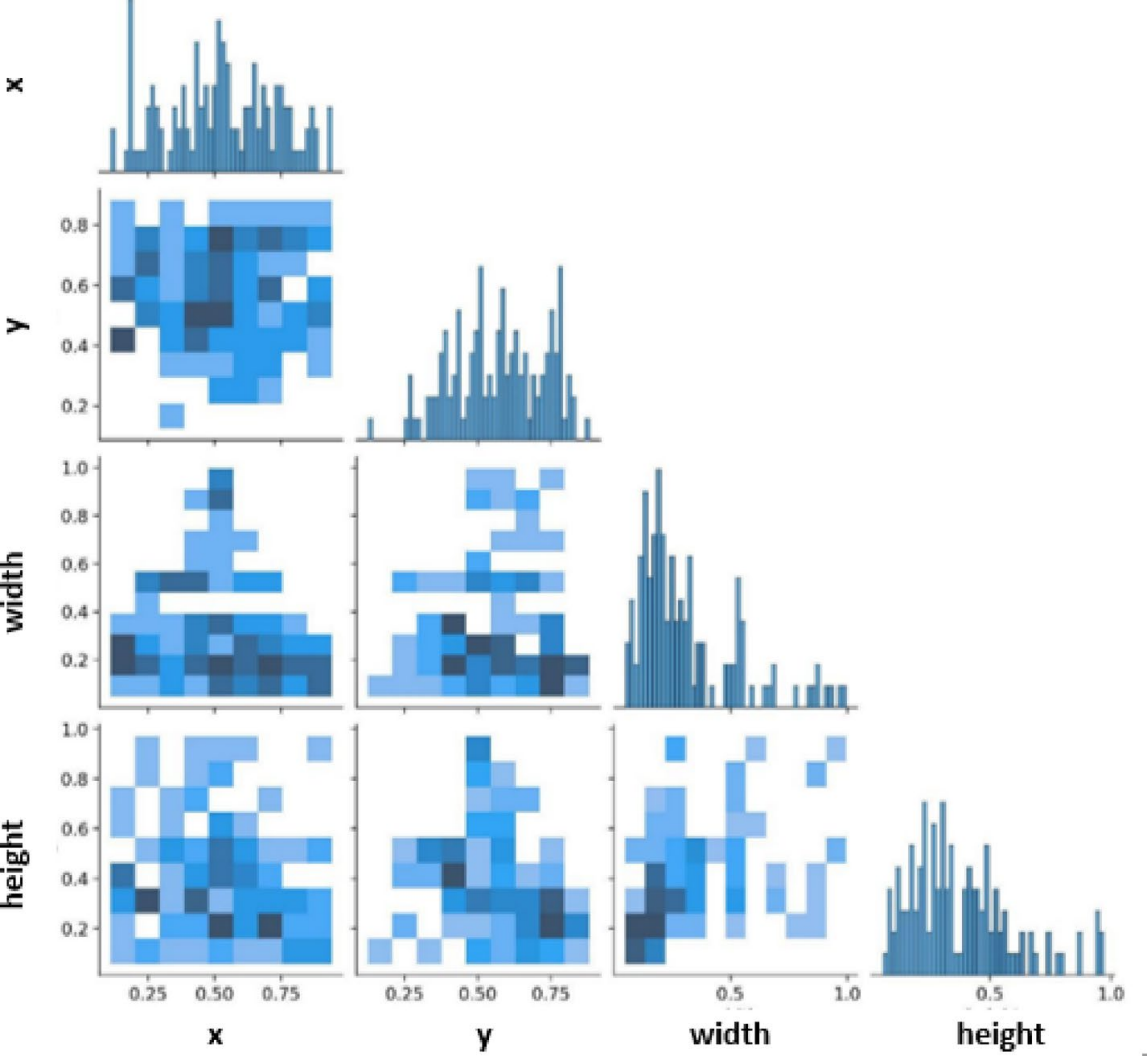
Labels Correlogram.

The monotonic relationship between Spearman’s rank correlation coefficient determines the monotonic relationship between the two variables Spearman’s correlation coefficient (*r*_*s*_).

2 $${r_s}=1 - 6\sum {\frac{{{d^2}}}{{n\left( {{n^2} - 1} \right)}}}$$

where d is the difference between the ranks of the two variables, and n is the number of observational pairs. Once we have calculated the correlations between the labels using one of the two measures, we can plot them in a matrix or grid to create a label correlogram. Then, we have the precision-confidence curve, a graphical representation of the relationship between precision and the confidence threshold of a machine learning model. Figure 11 illustrates the label correlogram. Here, each cell in the matrix represents the correlation between a pair of labels, and the cells are color-coded to indicate the strength of the correlation. The correlogram is a valuable method for examining the unpredictability of a data set. If the data were randomly generated, the autocorrelations for all time-lag separations should be near zero. At least one autocorrelation will be visibly non-zero if the data are not random. Correlograms are also utilized in the autoregressive moving average time series models’ identification phase. So, it is an excellent tool for detecting such unpredictability. The main applications are to check the data arbitrarily and whether an observation relates to an adjacent observation.

The precision of a model is expected to be high, and it indicates how accurate or inaccurate the prediction is across all classes^41^-^42^. Precision is a measure of the accuracy of its positive predictions. The following equation is used to calculate the precision at a particular confidence threshold:

3 $$\Pr {\text{ecision}}=\frac{{{{\text{T}}_{\text{P}}}}}{{{{\text{T}}_{\text{P}}}+{{\text{F}}_{\text{P}}}}}$$

**Fig. 12:**
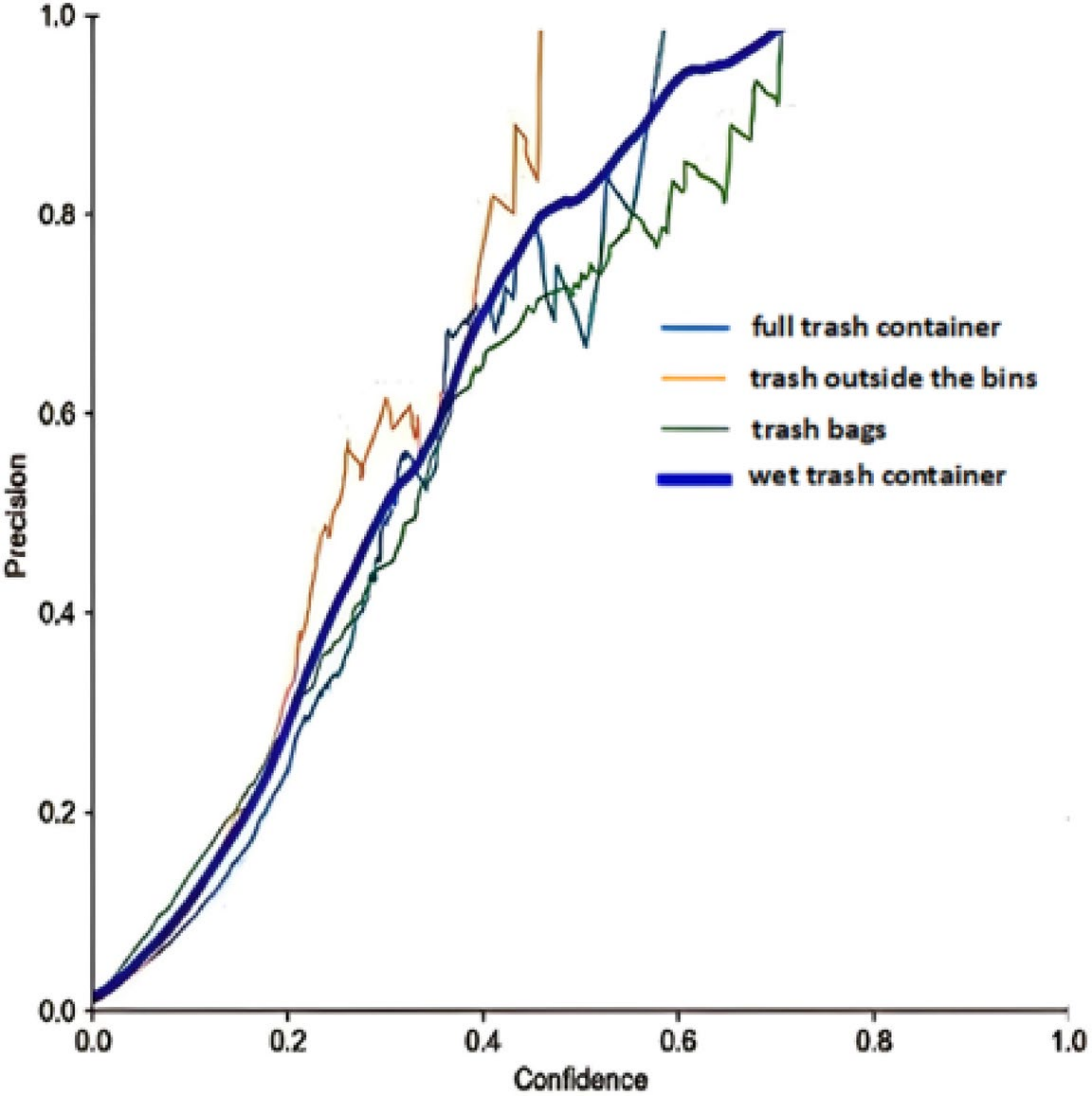
Precision Confidence Curve.

**Fig. 13:**
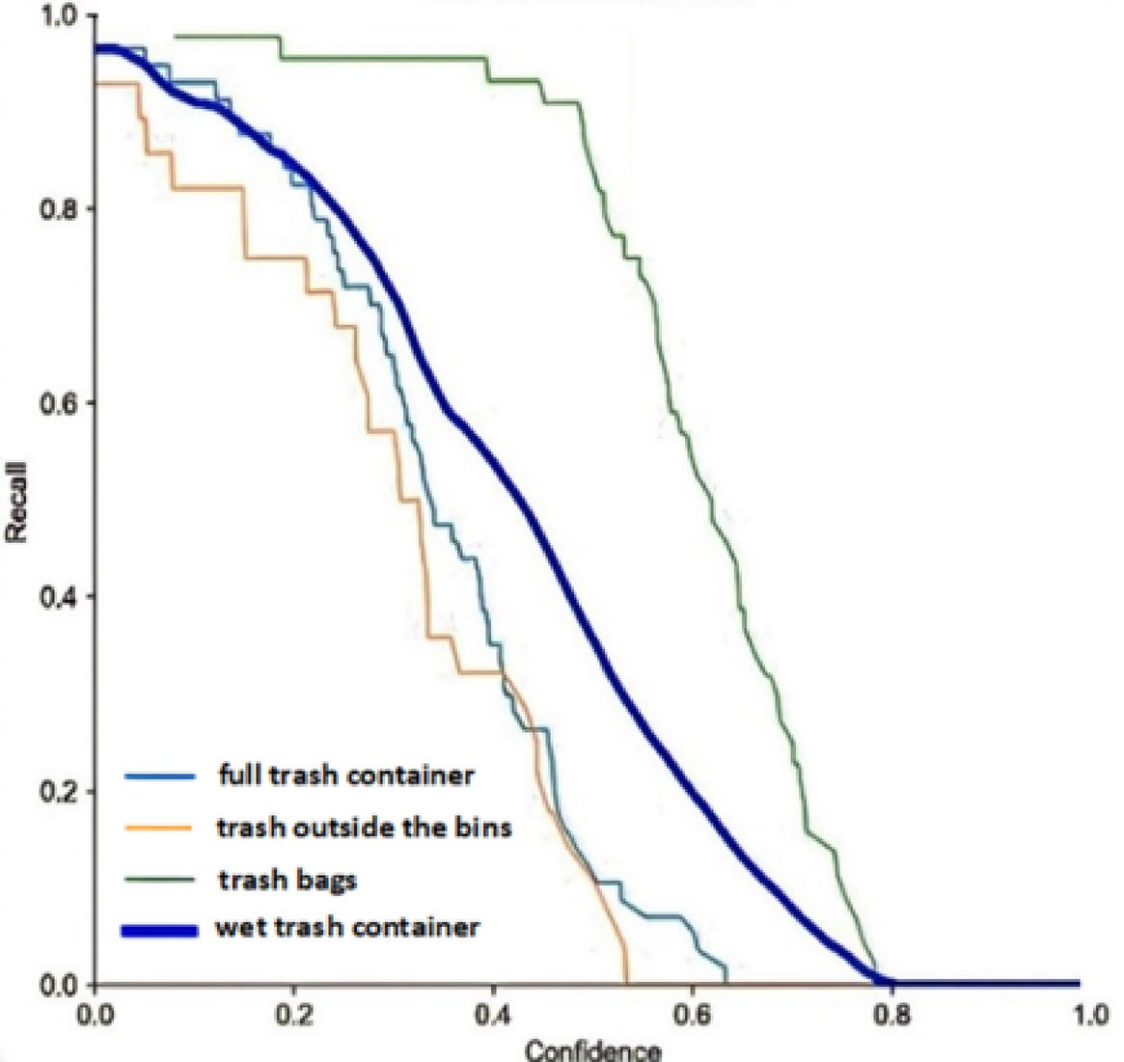
Recall Confidence Curve.

The number of examples the model correctly identified as positive is denoted by True Positives (TP), and the number of examples incorrectly classified as positive is denoted by False Positives (F_P_). The precision-confidence curve is drawn to understand the trade-offs between precision and the number of model positive predictions. The confidence threshold changes the precision as the model becomes more conservative in its predictions. This can help find the optimal balance between precision and the number of positive predictions for the dedicated application. The precision-confidence curve is shown in Fig. 12. It indicates the trade-offs between precision and the number of model-positive predictions. Similarly, the recall of a model at a particular confidence threshold is calculated using the following equations:

4 $$\:\text{R}\text{e}\text{c}\text{a}\text{l}\text{l}\:=\frac{\text{T}\text{P}}{\text{T}\text{P}+\text{F}\text{N}}$$

where the number of times the model is misclassified as negative is referred to as the False Negative (FN) rate. Moreover, the recall-confidence curve is a graphical representation of the relationship between the recall and the confidence threshold of a machine learning model. This curve helps understand the trade-offs between recall and the number of positive predictions a model makes. Changing the confidence threshold allows us to see how the recall changes as the prediction model becomes more conservative. This can help find the optimal balance between recall and the number of positive predictions for smart garbage monitoring and efficient garbage collection applications. It clarifies the trade-offs between precision and recall in a classification model. By changing the recall value, you can see how precision changes as the model becomes more complete in its positive predictions. This helps to find the optimal balance between precision and recall. Figure 13 illustrates an example of a recall-confidence curve.

**Fig. 14:**
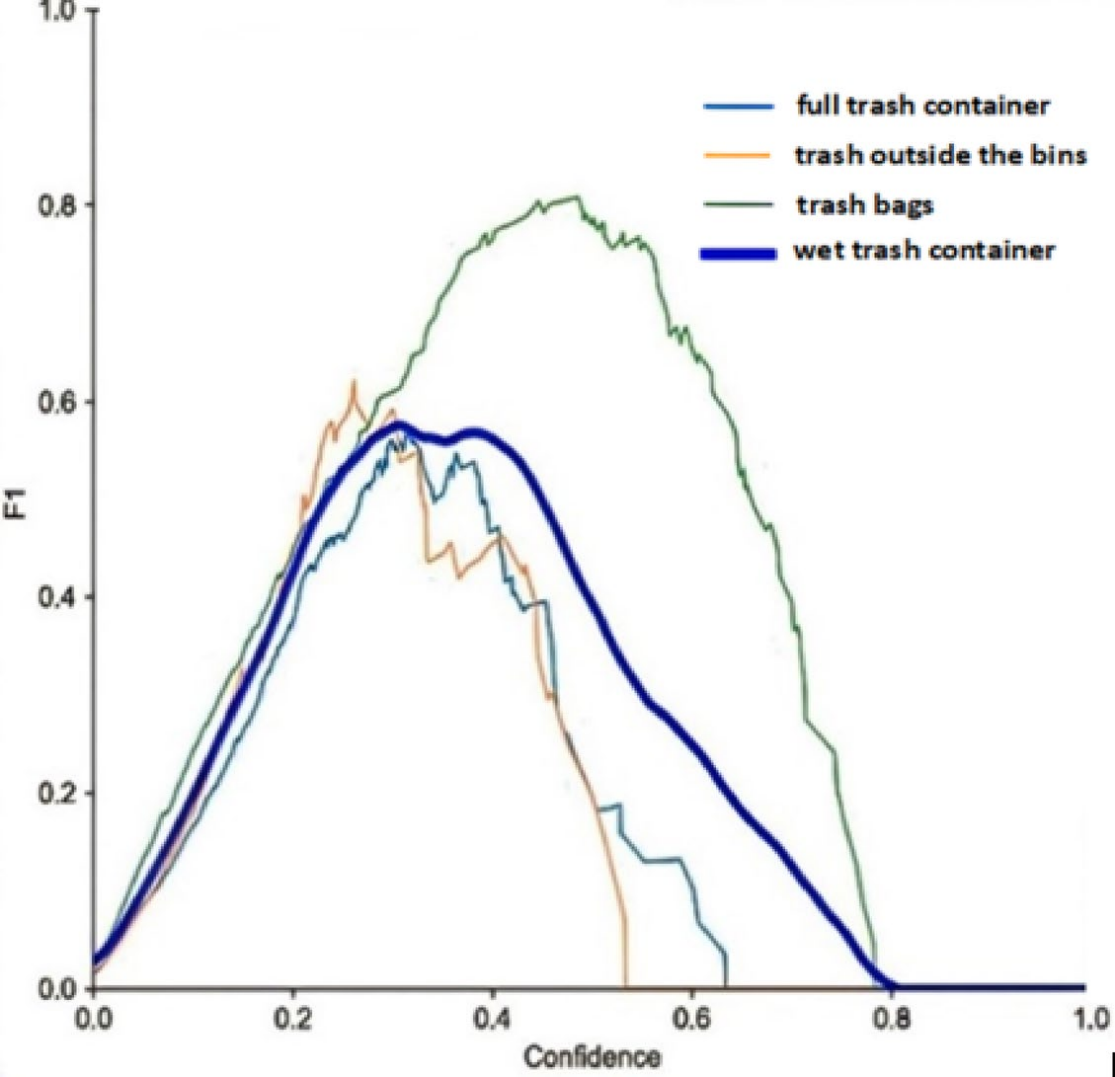
F1- Confidence Curve.

**Fig. 15:**
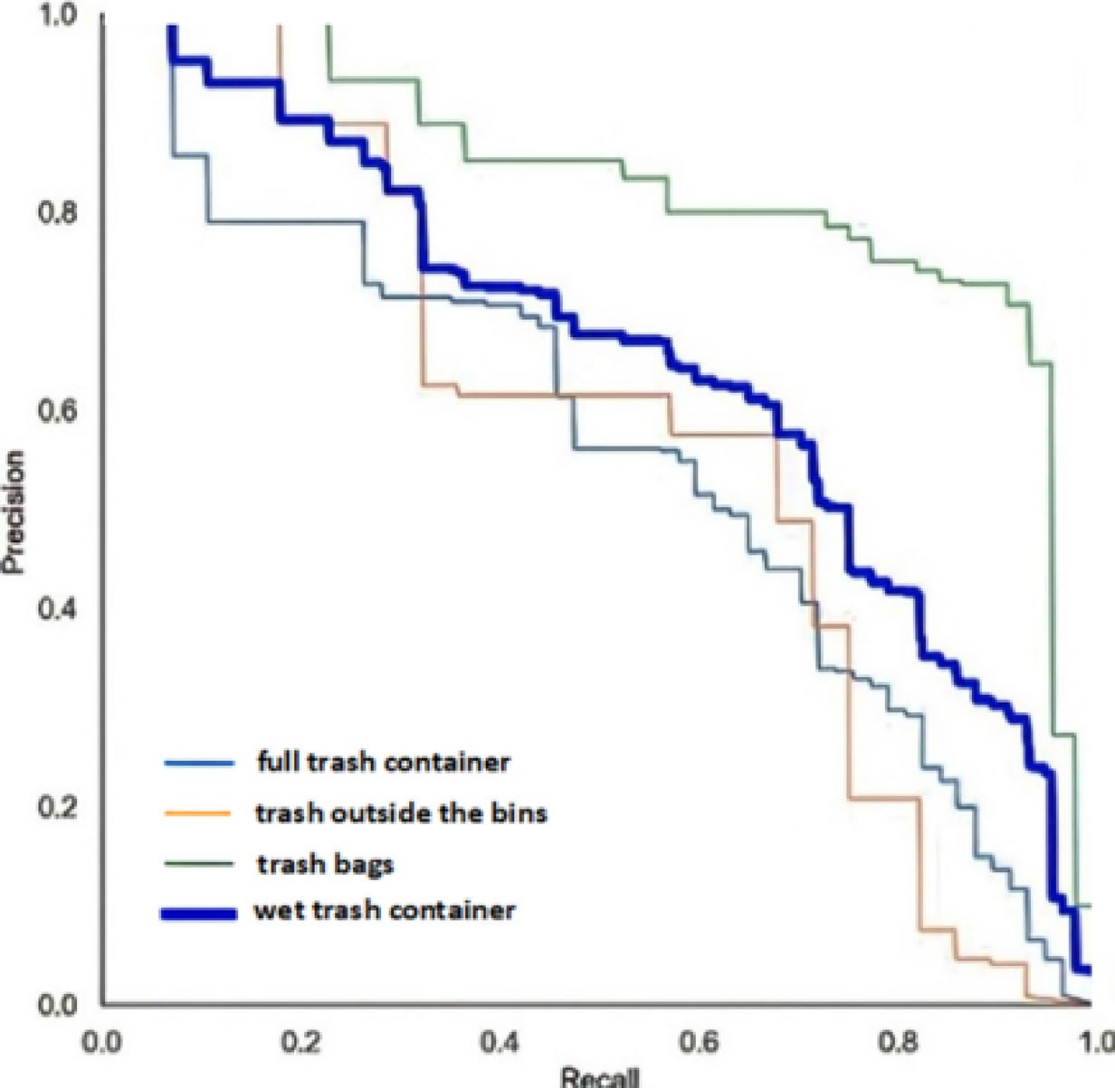
Precision-Recall Curve.

The F1 score is a valuable metric that balances precision and recall but should not be used in isolation, as it does not account for true negatives. This metric is a popular performance measure for classification and is typically preferred over accuracy. For example, it is used when data is unbalanced, i.e., when the number of samples belonging to one class substantially outnumbers those found in the other. Figure 14 illustrates the F1 confidence curve for the developed dataset. This figure shows that the F1 score helps identify the ideal confidence that balances the precision and recall values for the Yolov8 model. A model’s total performance can be assessed using the F1 score, which ranges from 0 to 1, with one being the best. So, it can be interpreted as the model’s balanced ability to capture positive cases (recall) and be accurate with the issues it does capture (precision) to give additional precision. The average precision (*AP*) is the most common object detection performance index, which is defined as the area under the precision-recall curve, calculated as follows:

5 $$\:AP={\int\:}_{0}^{1}Precision\left(r\right)\:dr$$

where $\:Precision\left(r\right)\:$ stands for the precision as a function of the recall (*r*). This curve shows the relationship between the precision and recall of a machine-learning model. Figure 15 illustrates the precision-recall curve.

The mean average precision (*mAP*) is also used to assess the average measurement accuracy in multi-class object identification. It is equal to the mean of the AP sums for all detection classes. As a result, it is frequently referred to as the detection model’s overall performance. The following equation can be used to get the *mAP*.

6 $$\:mAP\:=\frac{1}{\:{N}_{C}}\:\:\sum\:_{k=1}^{\:{N}_{C}}{AP}_{k}\:,\:$$

where *AP*_*k*_ is the *AP* of the *kth* object class, and *N*_*C*_ refers to the number of object classes. The $\:\text{F}1\:\text{s}\text{c}\text{o}\text{r}\text{e}$ is used to assess the performance of a classifier. The harmonic meaning of the model’s precision and recall is used to compute the F1 score. It represents the sensitivity and precision of the harmonic meaning. The F1 score of a model at a particular confidence threshold can be calculated using the following equation:

7 $$\:\text{F}1\:\text{s}\text{c}\text{o}\text{r}\text{e}\:=2*\frac{\text{P}\text{r}\text{e}\text{c}\text{i}\text{s}\text{i}\text{o}\text{n}\:\text{*}\:\text{R}\text{e}\text{c}\text{a}\text{l}\text{l}}{\text{P}\text{r}\text{e}\text{c}\text{i}\text{s}\text{i}\text{o}\text{n}\:+\:\text{R}\text{e}\text{c}\text{a}\text{l}\text{l}}$$

**Fig. 16:**
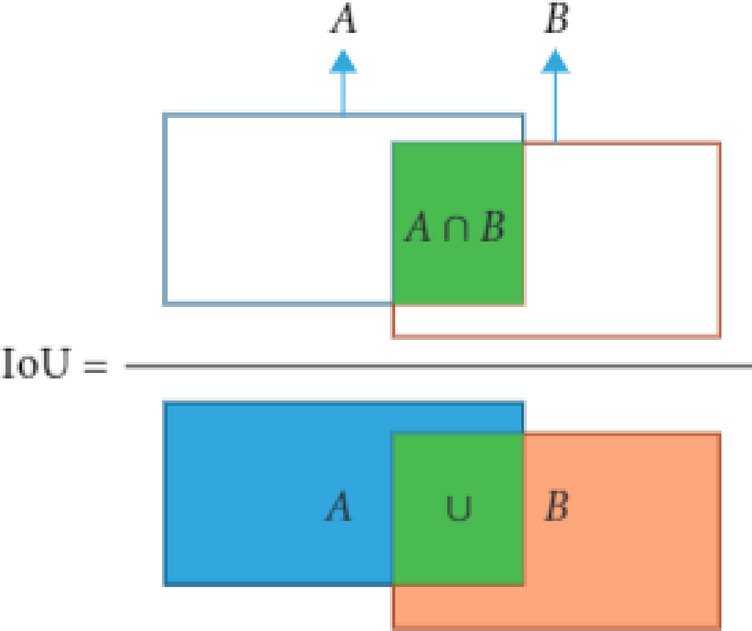
presents an IoU illustration.

Another method used as a performance indicator is the F1 confidence curve. A diagram shows the connection between a machine learning model’s confidence threshold and F1 score. The confidence threshold is the least expected probability a model must achieve to categorize a class as positive. Moreover, FPS is used to measure the model’s speed. It stands for frames per second, representing the number of images detected every second. When the FPS is greater than 30, it is regarded as real-time. The complexity of the model is measured by the floating-point operations (flops). Finally, an indicator of how well the predicted bounding box matches the ground truth box (the real object boundary) is called intersection over union (IoU). IoU is the ratio of the intersection and union areas of the predicted bounding boxes (B_p_) and the actual ground truth bounding box (B_gt_), as indicated in Fig. 16 and expressed by Eq. (8)^43^.

8 $$\:\text{I}\text{o}\text{U}\:=\:\frac{\text{a}\text{r}\text{e}\text{a}\:\left(\text{B}\text{p}\cap\:\text{B}\text{g}\text{t}\right)}{\text{a}\text{r}\text{e}\text{a}\:(\text{B}\text{p}\cup\:\:\text{B}\text{g}\text{t})}\:$$

Equation (8) describes one of the critical measures for assessing the precision of object detection algorithms. It has a value between 0 and 1 to describe the degree of overlap between the predicted and ground truth bounding boxes. A typical “good” IoU score is 0.5, whereas a theoretically ideal IoU score is 1. It aids in differentiating between “correct detection” and “incorrect detection.” IoU is also used to calculate mAP.

## Results and discussion

**Fig. 17:**
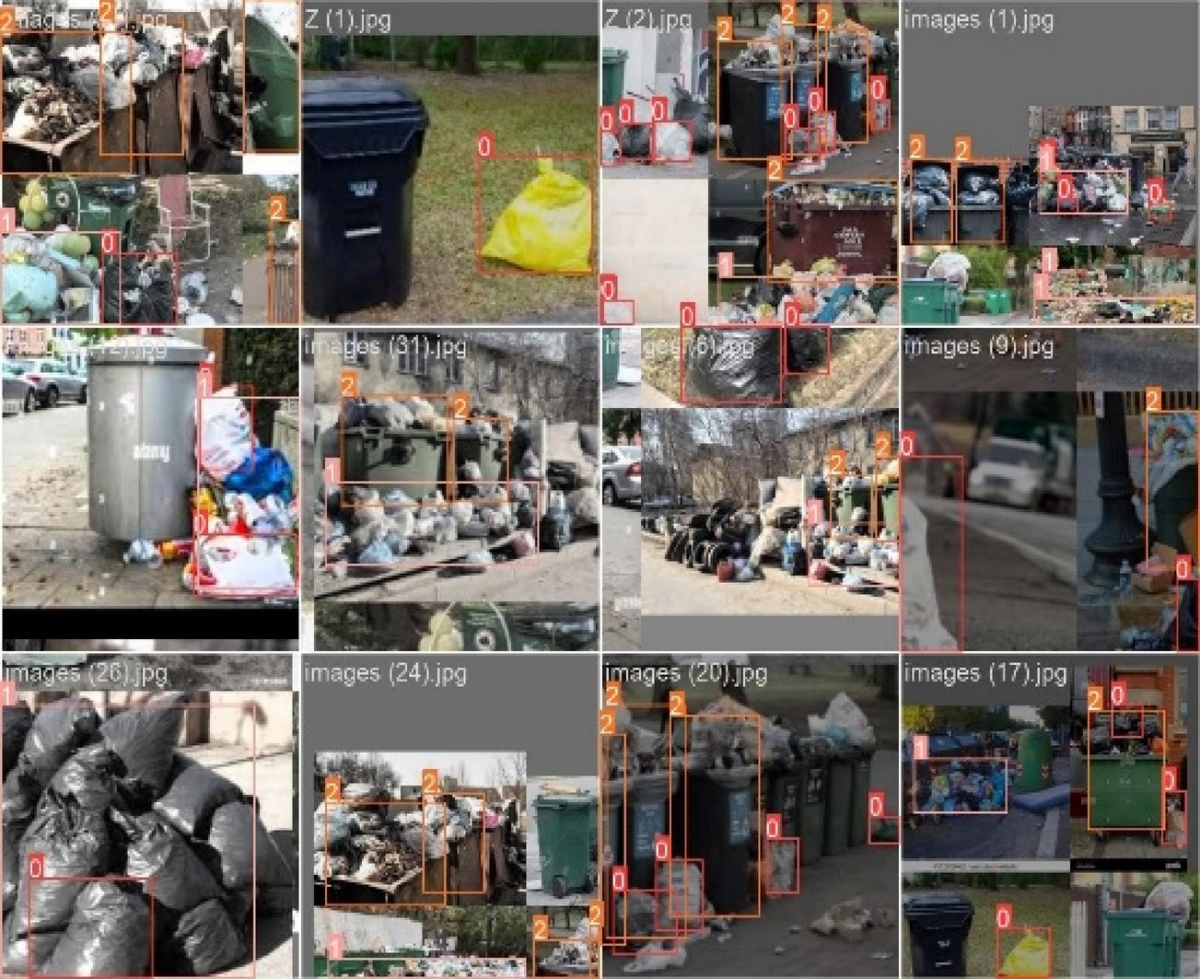
Samples of the collected images used as a dataset for training.

**Fig. 18:**
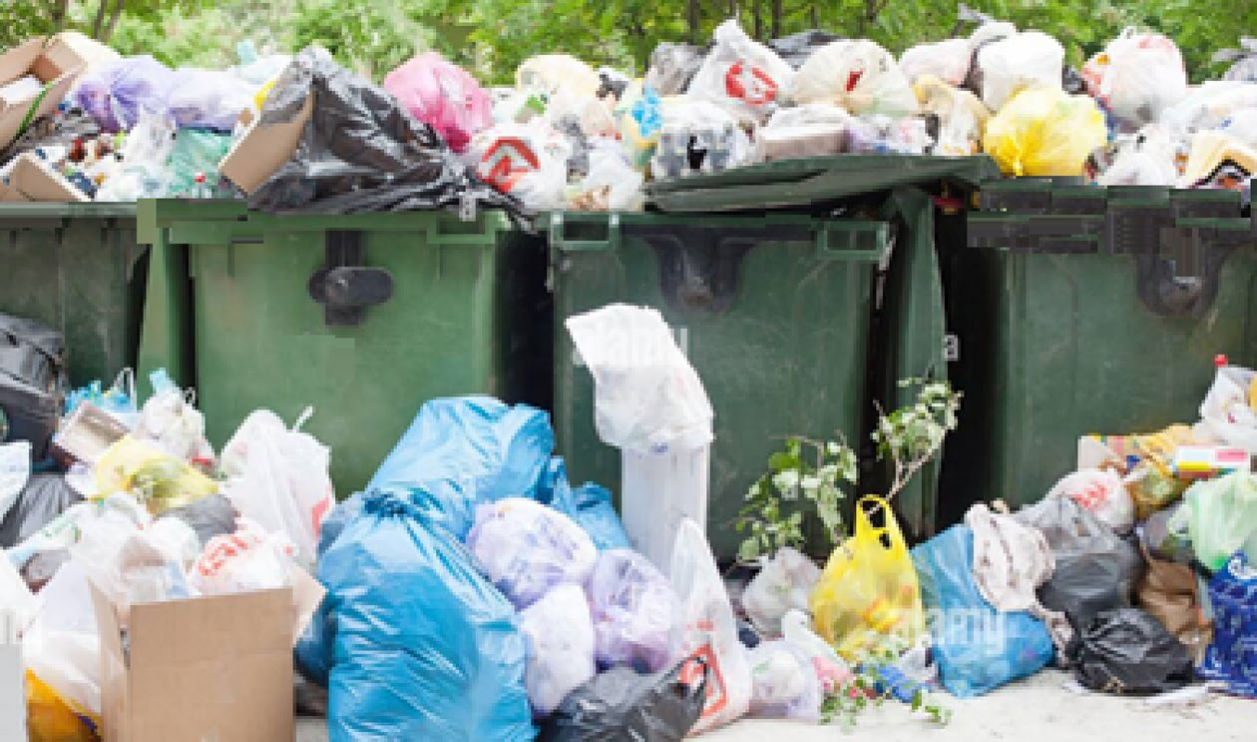
Test image.

This section compares the performances of the Yolov8 and Yolov5 models with those of the EfficientNet model. The three models are trained and tested on images of garbage datasets collected from the internet and garbage dumps. Samples of the gathered batches of images used as a dataset for training are shown on Fig. 17. This has been done by inserting the batches of images into the model and allowing it to detect different classes. Here, a transfer learning technique is adopted to fine-tune the deep-learning models learned on one task to do another related task. This method uses information from the pre-training task to enhance the model’s performance on the target task, frequently resulting in faster convergence and better outcomes. For the waste classification problem, the weights of Yolov8, Yolov5, and EfficientNet models are pre-trained on the ImageNet dataset, which comprises millions of images and thousands of object categories, to learn general features and representations of images. The adopted training epochs are 50, the batch size is 16, and the input image dimensions are 224 × 224. During each epoch, we tracked the training loss and accuracy, as well as the validation loss and accuracy, to evaluate the model’s performance and generalization capabilities. The chosen optimization method is stochastic gradient descent (SGD), employing a learning rate set at 0.01 and a momentum of 0.95. Figure 18 illustrates the test image used for testing the algorithm. The image contains four full trash containers, two trash bags, five trash outside the bins, and two wet trash containers.

### The findings using the created dataset

**Fig. 19:**
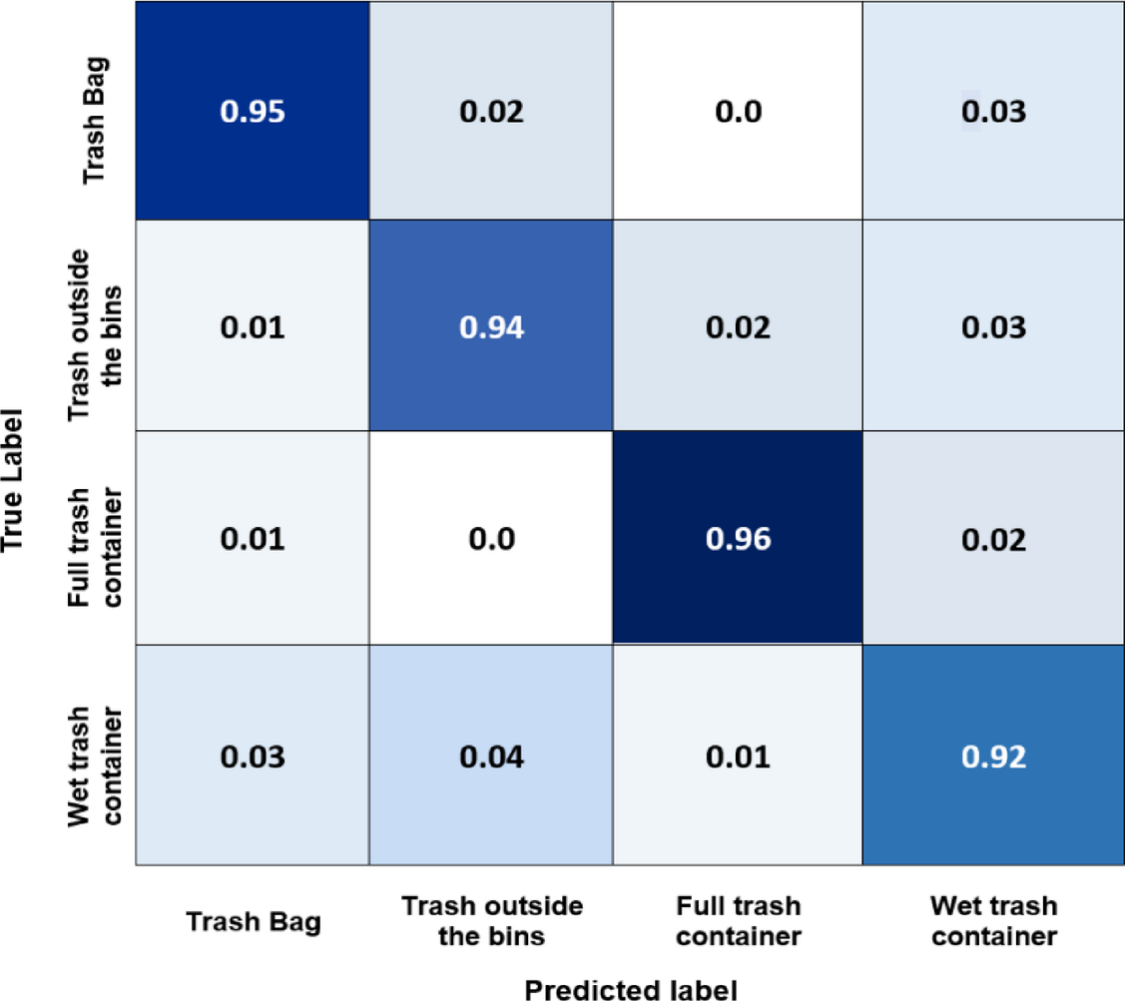
Normalized confusion matrix of Yolov8 for the four classes of the created dataset.

**Fig. 20:**
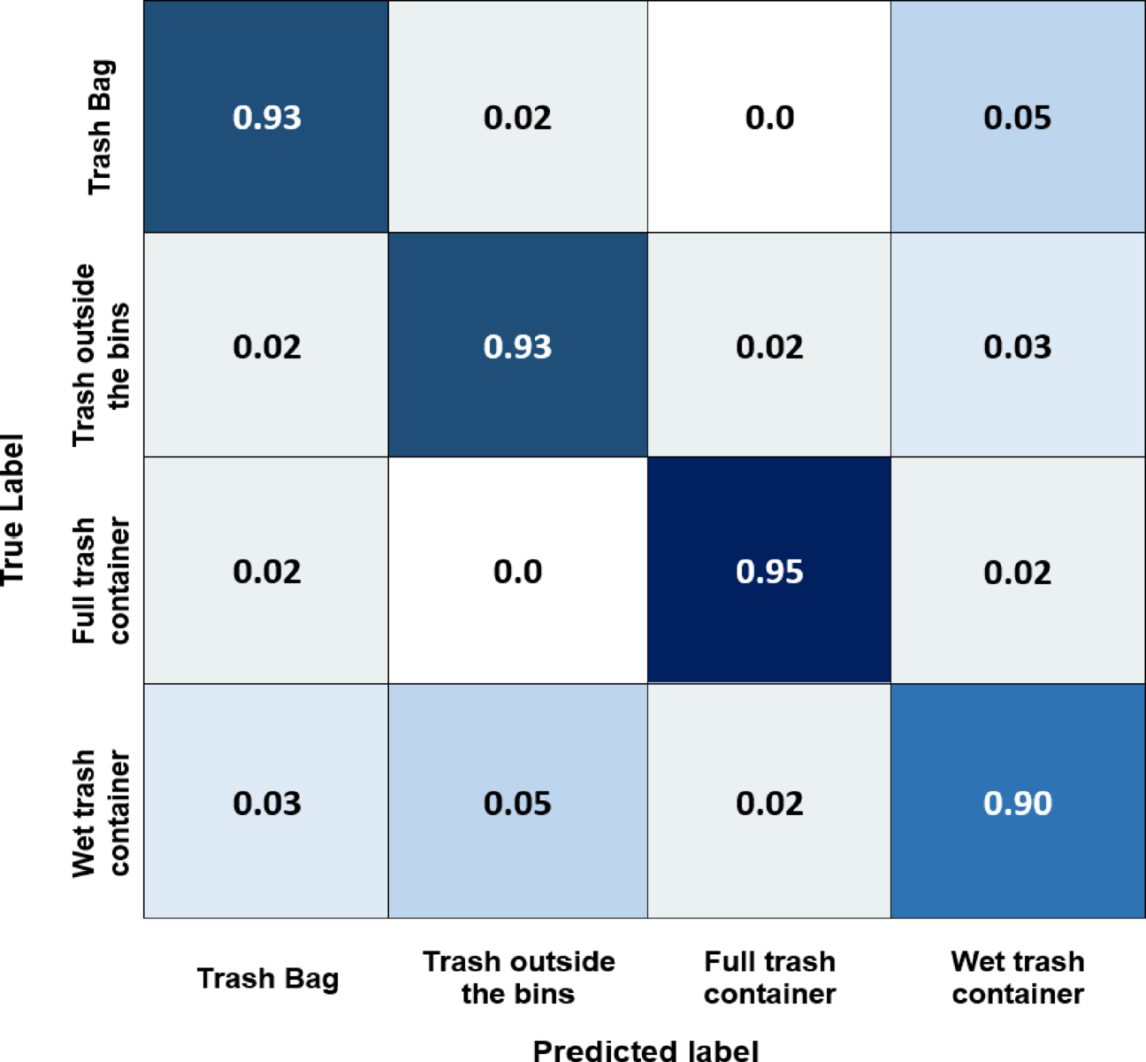
Normalized confusion matrix of Yolov5 model for the four classes of the created dataset.

**Fig. 21:**
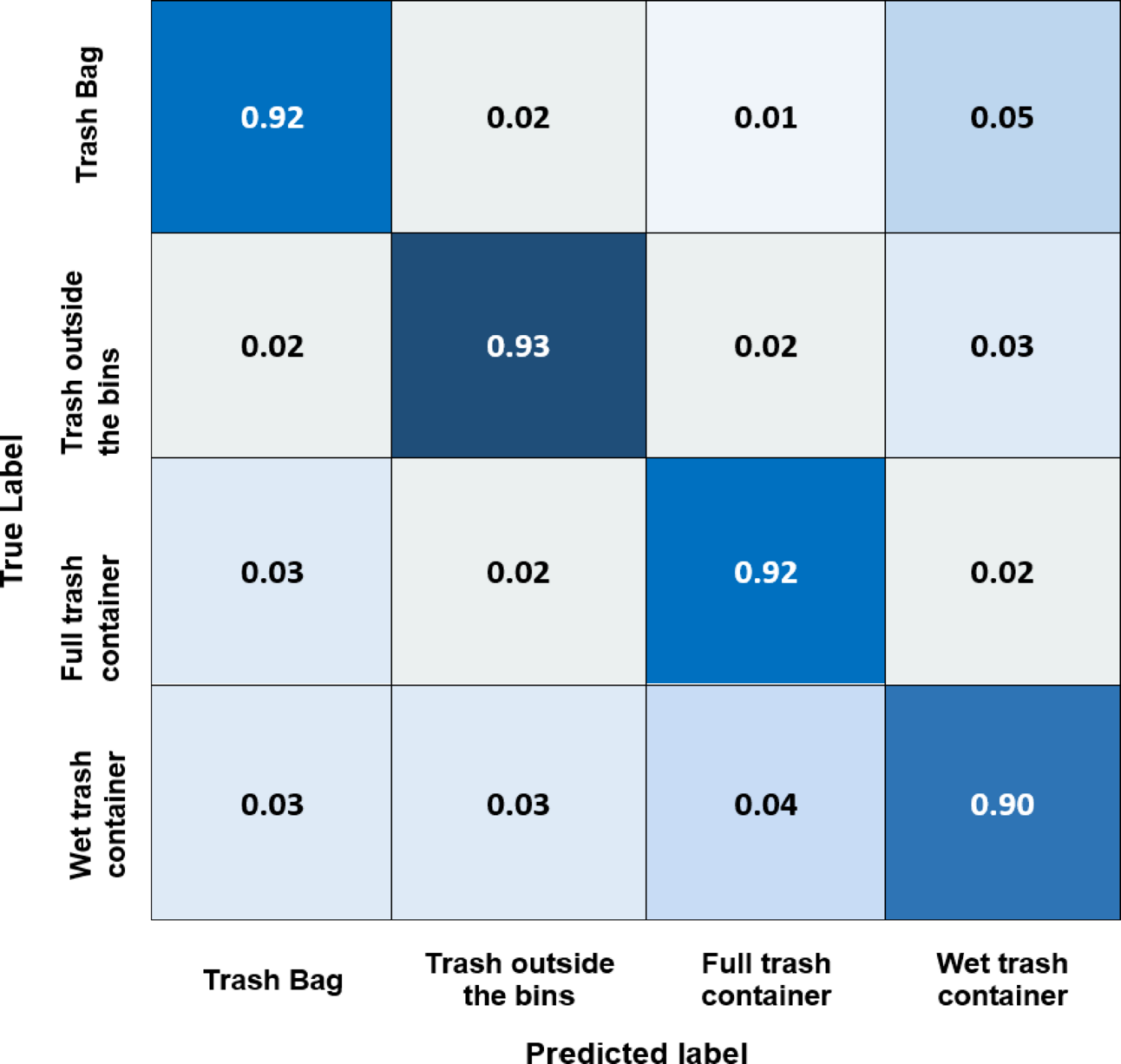
Normalized confusion matrix of EfficientNet model for the four classes of the created dataset.

Here, we demonstrate the main findings of adopting the created dataset detailed in Section IV.2. The confusion matrix facilitates evaluating a model’s performance for full trash containers, trash outside the bins, trash bags, and wet trash container classes. The cells in the table contain the count of examples that were predicted to have a particular label and have that label. Figures 19 and 20, and 21 illustrate the confusion matrix of the Yolov8, Yolov5, and EfficientNet models for different main classes of the gathered dataset. The models focus on the full trash bin class since it has the highest precision compared to other classes. This happened because of the model’s need for a larger dataset with more images representing the classes’ various sizes and shapes. If the model detects a full trash bin, it will send it to the predefined WhatsApp number of the person responsible for collecting the garbage. If the model detects a trash bag next to the garbage bin but not inside, an alarm will play to put the garbage bag inside.

Finally, the worst-case scenario is when someone suddenly drops a large amount of trash; the model will send it to authorities and send a photo of the incident. The confusion matrix for the Yolov8 model shows how well it performs across different classes. From Fig. 19, it can be deduced that the Yolov8 model has the best accuracy for most classes, with the full-trash bin class having the highest accuracy (0.96) and the wet trash container class having the lowest (0.92). These accuracies spotlight the degree to which the algorithm can categorize examples of various waste material types. This shows that this model is very good at differentiating between different trash categories. Figure 20 indicates that the Yolov5 model has good accuracy for most classes, with the full-trash bin class having the highest accuracy (0.95) and the wet trash container class having the lowest (0.90). With potential improvement in categorizing full trash bin and trash bag objects, the EfficientNet model performs well across most classes, according to the confusion matrix analysis performed on this 4-class dataset, as shown in Fig. 21. Table 6 includes the predicted labels and confusion matrix comparisons for the three models applied to the created dataset.

**Table 6:** Confusion matrix comparisons for the three models applied to the created dataset.

Model	Trash Bag	Trash outside bins	Full trash container	Wet trash container
Yolov8	0.95	0.94	0.96	0.92
Yolov5	0.93	0.93	0.95	0.90
EfficientNet	0.92	0.93	0.92	0.90

### Findings using the public dataset

**Fig. 22:**
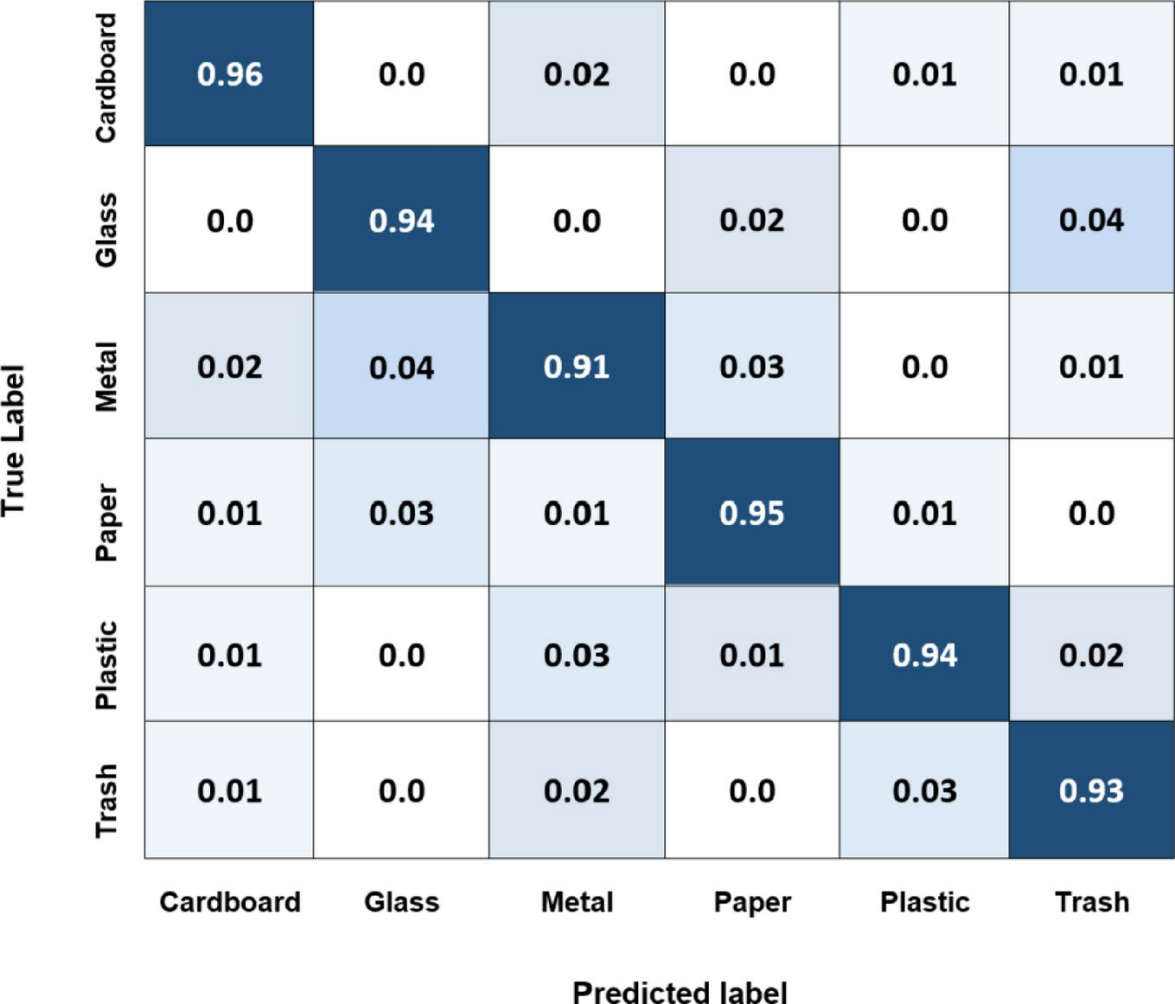
Normalized confusion matrix of the Yolov8 model for the six classes of the TrashNet dataset.

**Fig. 23:**
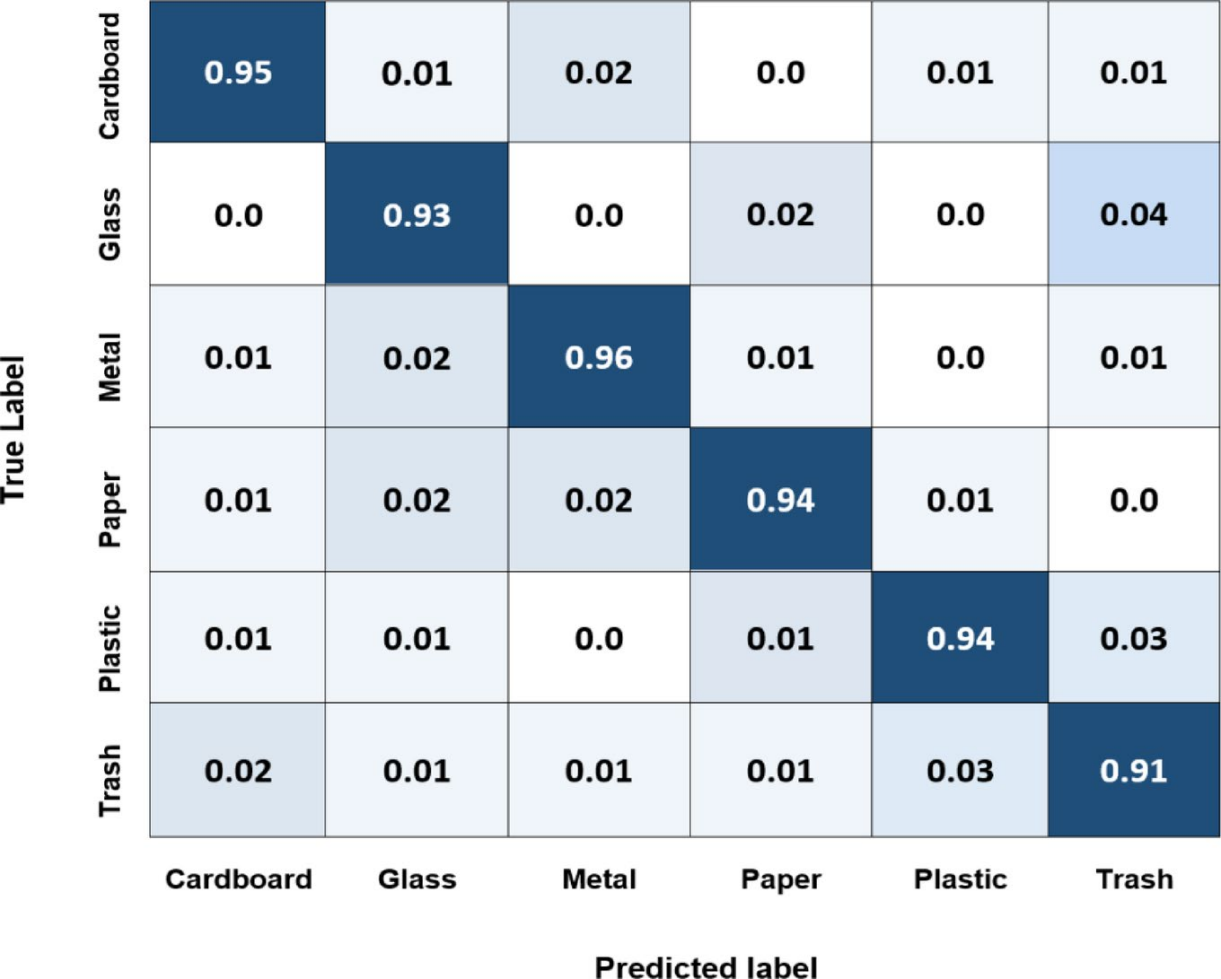
Normalized confusion matrix of the Yolov5 model for the six classes of the TrashNet dataset.

**Fig. 24:**
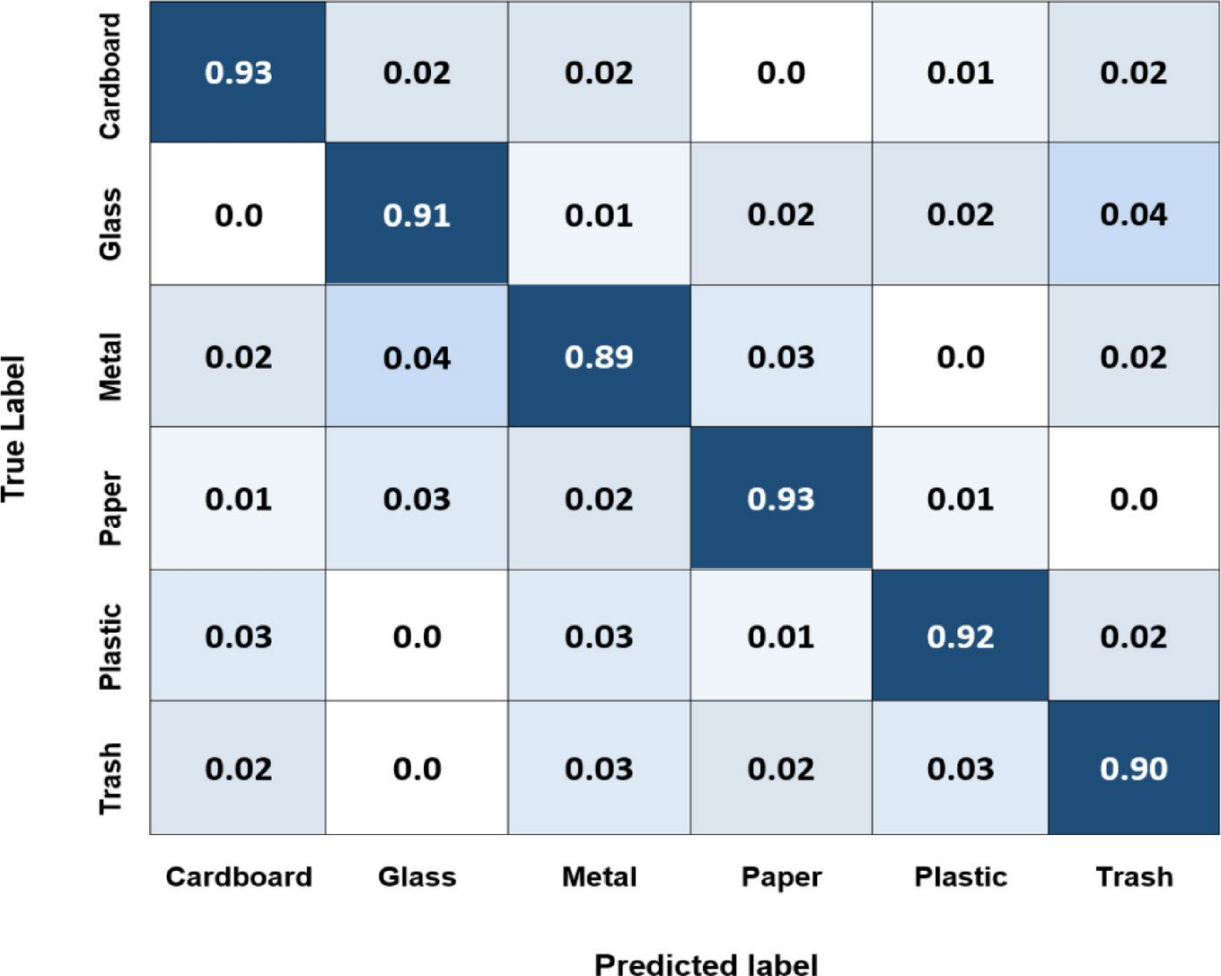
Normalized confusion matrix of the EfficientNet model for the six classes of the TrashNet dataset.

This subsection illustrates the capability of the proposed system to efficiently and accurately classify and sort complex waste materials in real-time. Two-stage classification strategies can refine the system, resulting in more precise waste classification and enhanced sorting outcomes. In addition, integrating this system with robotics will be investigated in future work to improve the sustainability and effectiveness of waste management. The system receives an image of a single piece of waste and produces a vector with probabilities for several categories. It detects objects along with the bounding boxes, which ensures the object detection algorithm only identifies each object once. Yolov8 and Yolov5 simultaneously predict multiple bounding boxes and class probabilities for those boxes. Here, Yolov8, Yolov5, and EfficientNet deep learning models are examined to test the object classification and recognition performances using the TrashNet dataset public dataset. Every model has advantages and disadvantages, and its performance varies according to the task and application. As shown in Fig. 22, the diagonal elements of the Yolov8 confusion matrix for each class, normalized by the total number of occurrences of that class, have values of 0.96, 0.94, 0.91, 0,95, 0.94, and 0.93, respectively. These values indicate how accurately the model identified each corresponding class. For instance, the cardboard class result of 0.96 means that 96% of all instances that correspond to this class have been identified correctly by the model. Figures 22 and 23, and 24 illustrate the confusion matrix of the Yolov8, Yolov5, and EfficientNet models for the six classes of the TrashNet dataset. Table 7 includes the three models’ predicted labels and confusion matrix comparisons. The Yolov5 model exhibits great accuracy for most classes, with the metal class and cardboard class exhibiting the highest accuracy (0.96 and 0.95, respectively). But the lowest for the trash category (0.91). This demonstrates the model’s ability to distinguish between various waste materials; however, it might have difficulty categorizing rubbish objects. For cardboard and paper, the EfficientNet model achieved the best classification accuracy score of 0.93, but it achieved the lowest score of 0.89 for metal. These numbers spotlight how well the model can categorize instances of various waste material types.

**Table 7:** Confusion matrix comparisons for the three models applied to the TrashNet dataset.

Model	Cardboard	Glass	Metal	Paper	Plastic	Trash
Yolov8	0.96	0.94	0.91	0.95	0.94	0.93
Yolov5	0.95	0.93	0.96	0.94	0.94	0.91
EfficientNet	0.93	0.91	0.89	0.93	0.92	0.90

The results demonstrated that Yolov5 and Yolov8 consistently outperformed EfficientNet in most waste classes. Yolov5 excels in wet trash container classification, whereas Yolov8 do well in full trash containers, trash outside the bins, and trash bag classification. Although EfficientNet performs poorly overall, it has the highest accuracy in the full-trash container category. The three models perform well in terms of precision, F1 score, precision, and recall. Yolov5 and Yolov8 have the highest precision, while EfficientNet achieves the best precision and recall. Yolov8 is the most complicated and time-consuming to train, whereas EfficientNet is the most efficient regarding the number of parameters. Yolov8 outperforms both Yolov5 and EfficientNet in terms of ROC values and mAP, as determined by the analysis. Consequently, Yolov8 was selected as the final model to be implemented in the proposed system to achieve high levels of accuracy in detecting and classifying the four distinct main classes. However, the problems here are the number of calculations and the speed.

## Future work and system limitations

The results show that the system has the advantage of solving the reliability problem of previously proposed systems to detect garbage if the trash bin is full or not, detect what is outside the trash bin, and miscommunication with the authorities to take necessary actions. Further research in this area might incorporate other data sources to improve the performance and accuracy of the model in subsequent works^44^. First, the data set employed a variety of trash types that it could not fully account for, and the situation of a garbage heap was not considered. The effect of using the same Yolo algorithm in both stages will also be investigated. The target occlusion detection scheme will be examined. In addition, the trash data set will be increased, and the deep learning models will be optimized, considering CPU and GPU capabilities to provide effective garbage collection. Moreover, further research into robotic integration with the proposed system can make waste management more effective and sustainable.

Dataset augmentation is crucial in deep learning, especially for tasks involving image classification and object detection. By increasing the diversity and size of the training dataset, the model’s robustness can be improved, leading to better generalization of unseen data. Specifying concrete augmentation techniques would strengthen the research. Specific augmentation strategies—such as applying more advanced methods like elastic deformation, random cropping, or color space adjustments provide a clear plan to increase dataset robustness by addressing challenges faced in waste classification. The rationale for selected augmentation methods illustrates the authors’ understanding of their dataset’s limitations and how they plan to overcome these hurdles. For example, if certain types of waste are underrepresented, the augmentation of these categories will improve the models’ performance.

The limitations of the proposed work on the intelligent garbage monitoring system can be highlighted as follows:

- The newly constructed garbage dataset may not encompass the full variety of waste types, including fewer common materials or shapes, potentially affecting the model’s generalizability and accuracy in diverse real-world situations.
- The models were developed and tested in controlled environments. Variations in environmental conditions, such as lighting changes, weather conditions (like rain or snow), and background clutter, can lead to detection errors when the models are deployed in unpredictable real-world settings.
- Although optimization has been made for running on Raspberry Pi and other embedded systems, the computational requirements for real-time image processing and analysis can still be demanding. There may be challenges in maintaining performance in areas with a high density of smart bins.
- The system’s effectiveness and reliability at a small scale are well-documented. However, there is a need for further validation regarding its performance when scaled up to cover a larger urban area or a more extensive network of bins.
- Integration with the existing waste collection infrastructure may present challenges, including compatibility with current technologies and systems used by waste management authorities.
- The proposed system relies on various sensors (e.g., for measuring waste levels and detecting waste outside the bins). These sensors may have limitations in accuracy or sensitivity that could impact detection performance.
- The effectiveness of the system depends on a reliable power supply. In areas where power outages are common, the continuous operation of the smart bins may be compromised.
- Although the system minimizes the need for human surveillance, it still requires human intervention to address issues such as maintenance of the bins, repairing sensors, or handling collected data, which may affect overall efficiency.
- The proposed system focuses primarily on real-time monitoring rather than predicting waste generation trends, which could enhance planning for collection operations.

## Conclusion

Effective waste management is crucial for improving urban living conditions using new technologies and advancements. This paper introduces real-time intelligent garbage monitoring and an efficient collection system using the Yolov5- and Yolov8-based models on the Raspberry PI to detect waste targets quickly and effectively. Waste classification is crucial to resolving the waste challenge and enhancing resource efficiency using a camera above the trash bin connected to a Raspberry Pi. A camera is employed to capture the image, and a GUI is developed to manage garbage collection in a user-friendly and configurable way. The paper proposes an enhanced accuracy real-time object detection system that promptly empties the garbage container to avoid the problem of trash around the bins and the amount of trash in them. If any individual throws garbage bags out of the bin or if the bin overflows, the system is triggered. The gadget will send the level with the given unique identity when it reaches the threshold so the responsible authorities can access these facts from their location and quickly clean up the trash cans. Also, a sensor above the trash is implemented to recognize the level of trash inside the container. When it reaches a specific limit, a notification is sent to the company responsible so that workers can take action to empty the basket. This research uses trash containers, bags, and dry and wet trash containers to separate waste. The trash is first classified into primary classes, each categorized into subclasses. To attain high levels of accuracy in identifying and categorizing the different types of waste products, Yolov5 is selected as the first stage. This achieves quick and precise results and addresses the demands of actual applications. It also reduces computing costs by considerably decreasing the number of parameters and reducing hardware requirements. So, the proposed algorithm is a better choice if detecting small objects on hardware without GPU capability is required. Each main class is further classified and broken down into sub-classes using Yolov8 to facilitate recycling. So, a real-time, lightweight, accurate garbage classification and detection system using deep learning models based on Yolov5 and Yolov8. The proposed system has shown significant improvements in accuracy and efficiency compared to traditional waste management approaches. For instance, the Yolov5 model achieved an impressive classification accuracy of 95% for detecting full trash bins and 90% for the wet trash container class, making it particularly efficient for applications requiring rapid deployment on devices with limited computational power. YOLOv8 demonstrates improved accuracy on complex waste detection tasks, achieving an accuracy of 96% for full trash bins, though it requires more significant computational resources. In contrast, the EfficientNet model showed higher accuracy (up to 93%) when classifying materials on public datasets but did not meet the precision levels of Yolov5 and Yolov8 for task-specific datasets. These numerical results highlight the efficacy of the proposed models in real-world applications compared to existing methods available in the literature.

## Data Availability

The datasets generated during the current study are not publicly available because the data have commercial value and maintain a competitive advantage but are available from the corresponding author on reasonable request.
